# Chaperone-mediated autophagy compensates for impaired macroautophagy in the cirrhotic liver to promote hepatocellular carcinoma

**DOI:** 10.18632/oncotarget.16685

**Published:** 2017-03-29

**Authors:** Srinivas Chava, Christine Lee, Yucel Aydin, Partha K. Chandra, Asha Dash, Milad Chedid, Swan N. Thung, Krzysztof Moroz, Tong Wu, Nabeen C. Nayak, Srikanta Dash

**Affiliations:** ^1^ Department of Pathology and Laboratory Medicine, Tulane University Health Sciences Center, New Orleans, Louisiana, USA; ^2^ Department of Medicine, Division of Gastroenterology and Hepatology, Tulane University Health Sciences Center, New Orleans, Louisiana, USA; ^3^ The Lillian and Henry M. Stratton-Hans Popper Department of Pathology, Icahn School of Medicine at Mount Sinai, New York, New York, USA; ^4^ Senior Consultant and Advisor, Sir Ganga Ram Hospital, Department of Pathology, New Delhi, India

**Keywords:** liver cirrhosis, hepatocellular carcinoma, macroautophagy, chaperone-mediated autophagy, endoplasmic reticulum, Pathology Section

## Abstract

Macroautophagy and chaperone-mediated autophagy (CMA) represent two major lysosomal degradation processes and often compensate for one another to facilitate cell survival. The aim of this study was to determine whether these autophagy pathways could compensate for one another to promote HCC cell survival in the cirrhotic liver. Analysis of normal liver tissue showed no expression of glypican-3 or p62 proteins, suggesting that macroautophagy is the major contributor to autophagic flux under non-pathological conditions. Of 46 cirrhotic livers with HCC examined, 39 (84%) of HCCs showed increased expression of p62, and 36 (78%) showed increased expression of glypican-3, while adjacent non-tumorous hepatocytes were negative for expression of p62 and glypican-3, similar to normal liver tissue. These results suggest that macroautophagy flux is impaired in HCC. Furthermore, more than 95% of HCCs showed altered expression of LAMP-2A compared to the surrounding non-tumorous cirrhotic liver, consistent with induction of CMA in HCC. Elevated expression of glucose-regulated protein 78 (GRP78) and heat shock cognate protein (Hsc70) were detected in 100% of HCC and adjacent non-tumorous cirrhotic livers, suggesting that unresolved ER-stress is associated with HCC risk in liver cirrhosis. Interestingly, inhibition of lysosomal degradation using hydroxychloroquine (HCQ) induced expression of the tumor suppressor p53, promoted apoptosis, and inhibited HCC growth, whereas activation of autophagy using an mTOR inhibitor (Torin1) promoted HCC growth. Results of this study suggest that induction of CMA compensates for the impairment of macroautophagy to promote HCC survival in the cirrhotic liver.

## INTRODUCTION

Hepatocellular carcinoma (HCC) is a primary malignant tumor in the liver that frequently develops in the background of pre-existing chronic liver diseases. While liver cancer is the fifth most common neoplasm worldwide, the poor prognosis associated with this disease makes it the third leading cause of cancer-related deaths [1]. HCC accounts for 80-90% of primary liver cancers, and the incidence of HCC is increasing globally by 3-9% annually [2]. Each year, more than half a million people are diagnosed with HCC worldwide, with nearly 20,000 new cases occurring in the United States [3]. The majority of HCCs develop in patients with liver cirrhosis resulting from infection with either the hepatitis B virus (HBV) or the hepatitis C virus (HCV) [4]. Other conditions, such as alcoholic hepatitis, non-alcoholic fatty liver disease, diabetes, and hemochromatosis also contribute to the development of HCC [5, 6]. The incidence of HCC varies geographically, with the highest rates in East Asian countries and Africa [7]. Although HCC incidences in Europe and North America are moderate, rates in these regions continue to rise due to the obesity epidemic and other metabolic syndromes [8].

The most effective treatments for HCC require detection of the disease at early stages; HCC neoplasms detected at an early stage can be cured by liver transplantation, surgical resection, and/or percutaneous radiofrequency ablation [9]. Liver transplantation yields the highest survival rates for patients with HCC, but this therapeutic approach is limited due to a lack of donor organs [10]. Surgical resection and percutaneous ablation also show relatively high response and survival rates, although the long-term (10-year) survival rate is only 22%-35% [11]. Treatment options for advanced stage HCC are often ineffective. The survival rate of patients with advanced liver cancer is less than 12 months, underscoring the urgent need to develop effective therapeutic strategies to treat this disease [9]. Current treatments, such as transarterial chemoembolization (TACE), radiotherapy, and conventional FDA-approved chemotherapy with the multikinase inhibitor sorafenib, have shown limited success [12–14].

While our understanding of the molecular pathogenesis of chronic liver disease and liver cirrhosis has improved significantly over the last decade, the exact mechanism by which liver cirrhosis contributes to the formation of HCCs remains largely unknown. Current models suggest that HCCs develop in the cirrhotic liver through a multistep process, starting from low-grade and progressing to high-grade dysplastic nodules, and eventually HCC [15]. Genetic alterations in the hepatocytes of precancerous lesions occur due to accumulation of a wide spectrum of mutations, resulting in the activation of oncogenic signaling and malignant transformation [16]. However, no systematic study has been performed to explain why these transformative molecular alterations occur most often in liver cirrhosis.

Autophagy plays an important role in the liver and contributes to the evolution of chronic diseases induced by viral infections and alcohol abuse, as well as non-alcoholic fatty liver diseases, fibrosis, aging, liver ischemia-perfusion injury, and cancer. Increasing amounts of evidence indicate that alterations in pro-death to pro-survival pathways maintained by autophagy promote HCC development in the cirrhotic liver [17–19]. However, autophagy has been recognized as a process that is common to the pathogenesis of chronic liver disease, liver fibrosis, and HCC. We have previously shown that most HCCs express p62 at levels above the surrounding non-tumorous cirrhotic liver tissue, suggesting that macroautophagy is impaired in HCC. Macroautophagy (autophagy) and chaperone-mediated autophagy (CMA) are two well-characterized autophagy processes that occur in mammalian cells [20]. In the case of macroautophagy, aggregated proteins and/or damaged or modified organelles are sequestered in vesicles and degraded after fusion with the lysosome. This process is orchestrated by a set of autophagy-related proteins (ATGs) and depends on cellular mTOR activity [21]. In contrast, CMA selectively degrades cytosolic proteins in the lysosome without vesicle formation. Cytosolic proteins containing a pentapeptide amino acid motif (KFERQ) are targeted to the lysosome by direct interaction with the heat shock cognate protein 70 (Hsc70) [22]. The protein-Hsc70 complex binds to the lysosome membrane through an interaction with lysosome-associated membrane protein type 2A (LAMP-2A) and is subsequently translocated across the lysosomal membrane and degraded. LAMP-2A is one of the three splice variants of the LAMP2 gene and is a single-span membrane protein with a short 12-amino acid C-terminus tail that is exposed on the surface of the lysosome [23]. Interestingly, the number of LAMP-2A molecules directly correlates with CMA activity under different pathological conditions, and cells modulate CMA levels through up- or downregulation of LAMP-2A expression [23].

Previous studies have shown that under serum starvation, macroautophagy and CMA are activated sequentially, rather than simultaneously, suggesting that these two pathways are not completely independent [22, 23]. Furthermore, blockage or deficiency of one autophagy pathway may lead to the activation of the other [24, 25]. In serum-starved cells, protein degradation by vesicle-mediated autophagy switches to a CMA-mediated process to facilitate cell survival when macroautophagy slows. Crosstalk between these two forms of autophagy is essential for cell survival under conditions of viral infection and other types of cellular stress.

We and other researchers have reported that HCCs derived from human cirrhotic livers exhibit increased expression of p62, suggesting impaired macroautophagy flux in these neoplasms [26, 27]. However, the mechanisms by which HCC cells survive in the cirrhotic liver remain largely unknown. In this study, we sought to determine whether CMA compensates for impaired macroautophagy to promote HCC survival in the cirrhotic liver by examining the expression of p62 and LAMP-2A in HCC-positive cirrhotic liver tissue sections. We found increased expression of p62 and glypican-3 in most of the HCCs, but not in the non-tumorous cirrhotic livers, consistent with a decrease in macroautophagy in HCC. Altered expression of LAMP-2A was observed in more than 95% of the HCCs, suggesting that CMA is induced in HCC. Our results suggest that a decrease in macroautophagy with a concomitant increase in CMA activity is associated with progression of HCC in liver cirrhosis.

## RESULTS

### Impaired macroautophagy flux in HCC

**Figure 1:**
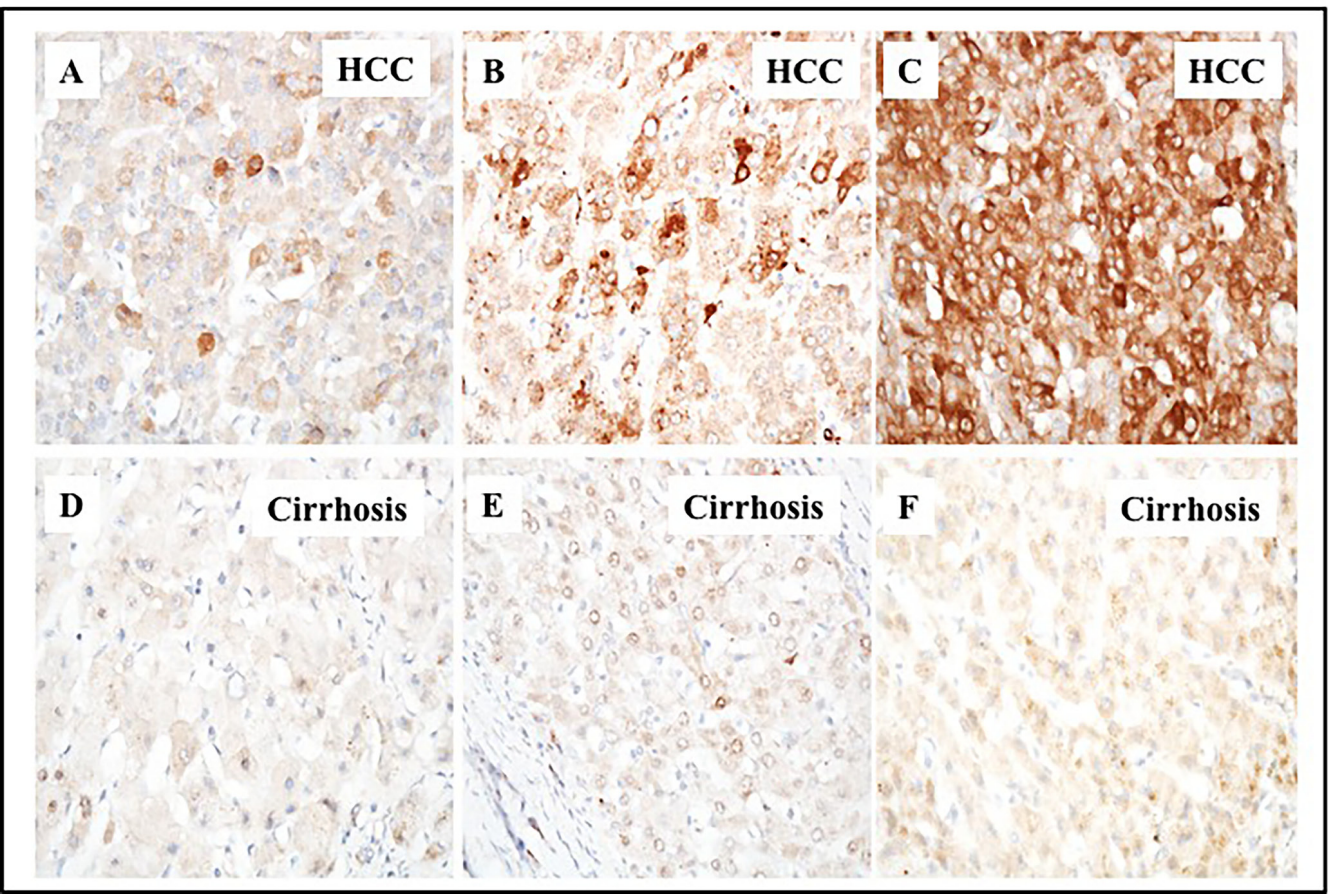
Immunohistochemical staining of p62 protein in HCC and the adjacent non-tumorous cirrhotic liver A representative picture showing the proportion of p62 staining immunopositive cells observed in cirrhotic livers with HCC. **A**. HCC with 1-10% cells are positive. **B**. HCC with 10-50% cells are positive. **C**. HCC with 50-100% cells are positive for p62 staining. **D**., **E**. and **F**. represents the negative staining seen in the adjacent non-tumorous tissues shown in upper panel **A**., **B**. and **C**. respectively. (Pictures were taken at 40 X magnification).

While liver cirrhosis is an independent risk factor for HCC development, the mechanisms that contribute to HCC development in the cirrhotic microenvironment are unknown. We sought to determine whether autophagy plays a role in HCC survival in the cirrhotic liver. Since p62 expression levels are inversely correlated with autophagic activity, we used semiquantitative immunohistochemistry to measure expression of p62 in HCC and adjacent non-tumorous cirrhotic liver tissues from 46 paraffin-embedded tissue sections [28]. We found that 84% (39/46) of the HCCs were positive for p62 expression. In contrast, none of the non-transformed hepatocytes in the cirrhotic surrounding area were positive for p62 expression, indicative of active macroautophagy flux in liver cirrhosis. Tissue sections from eight normal livers that had neither cirrhosis nor HCC also showed no evidence of p62 expression. Proportions of positive cells varied among the HCC samples, with 10-100% of cancerous cells showing p62 positive staining (Figure 1). Expression of p62 in HCC cells was localized mostly in the cytoplasm, with some HCC cells showing perinuclear localization of the protein.

**Figure 2:**
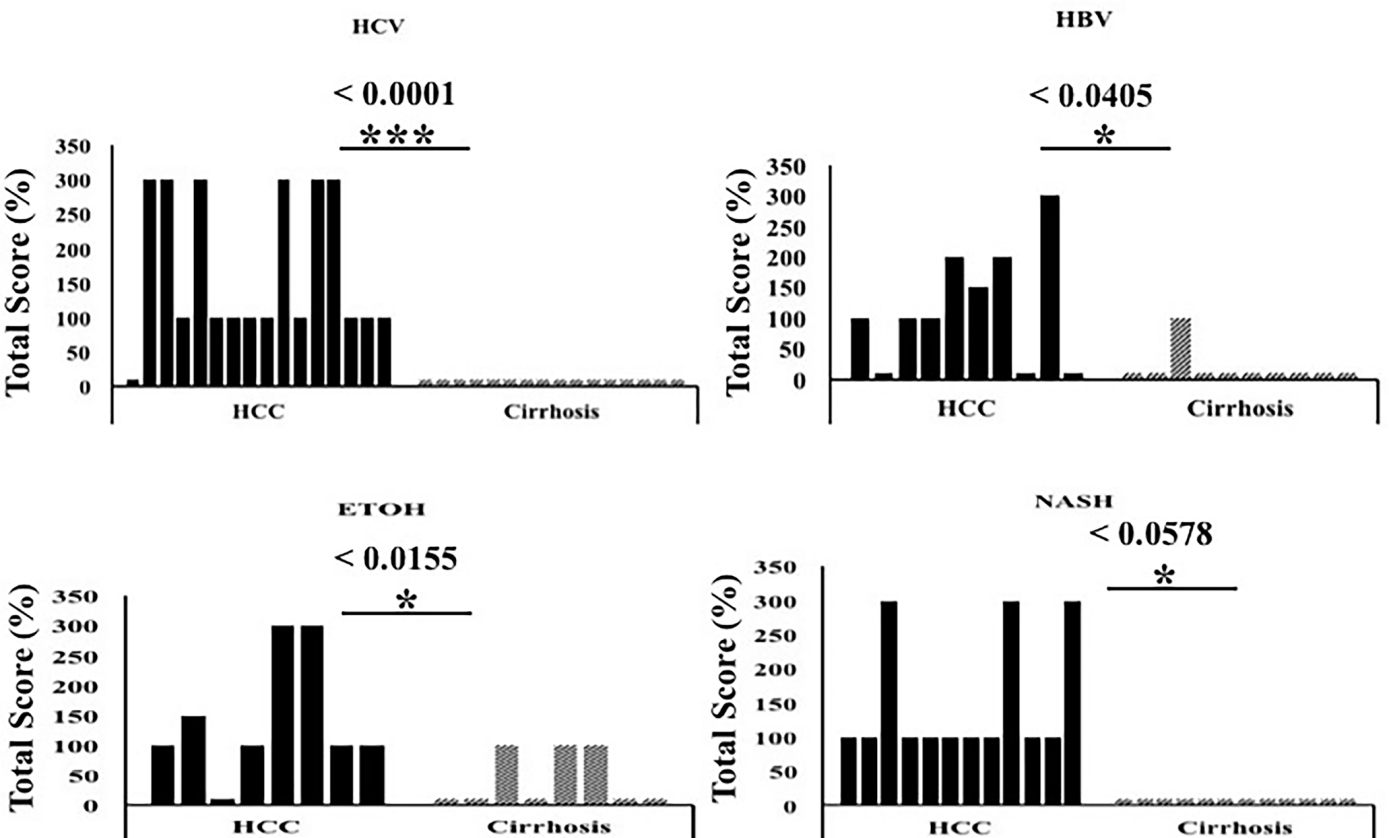
The expression of p62 between HCC and non-tumorous cirrhotic liver tissues of different etiologies Staining intensity was semiquantified by considering the intensity of staining and the proportion of immunopositive cells. By multiplying the staining intensity score and the proportion of immunopositive cells, a staining score of 0-300 was determined. HCC cases showed increased p62 staining (median intensity 200; range 0-300), compared to the corresponding non-tumorous tissue of the cirrhotic liver (median 0, range 0-300). The p62 staining was found to be significantly high in HCC of different etiologies as compared to the adjacent non-tumorous cirrhotic liver. * *P* < 0.05, ** *P* < 0.001 and *** *P* < 0.0001.

**Figure 3:**
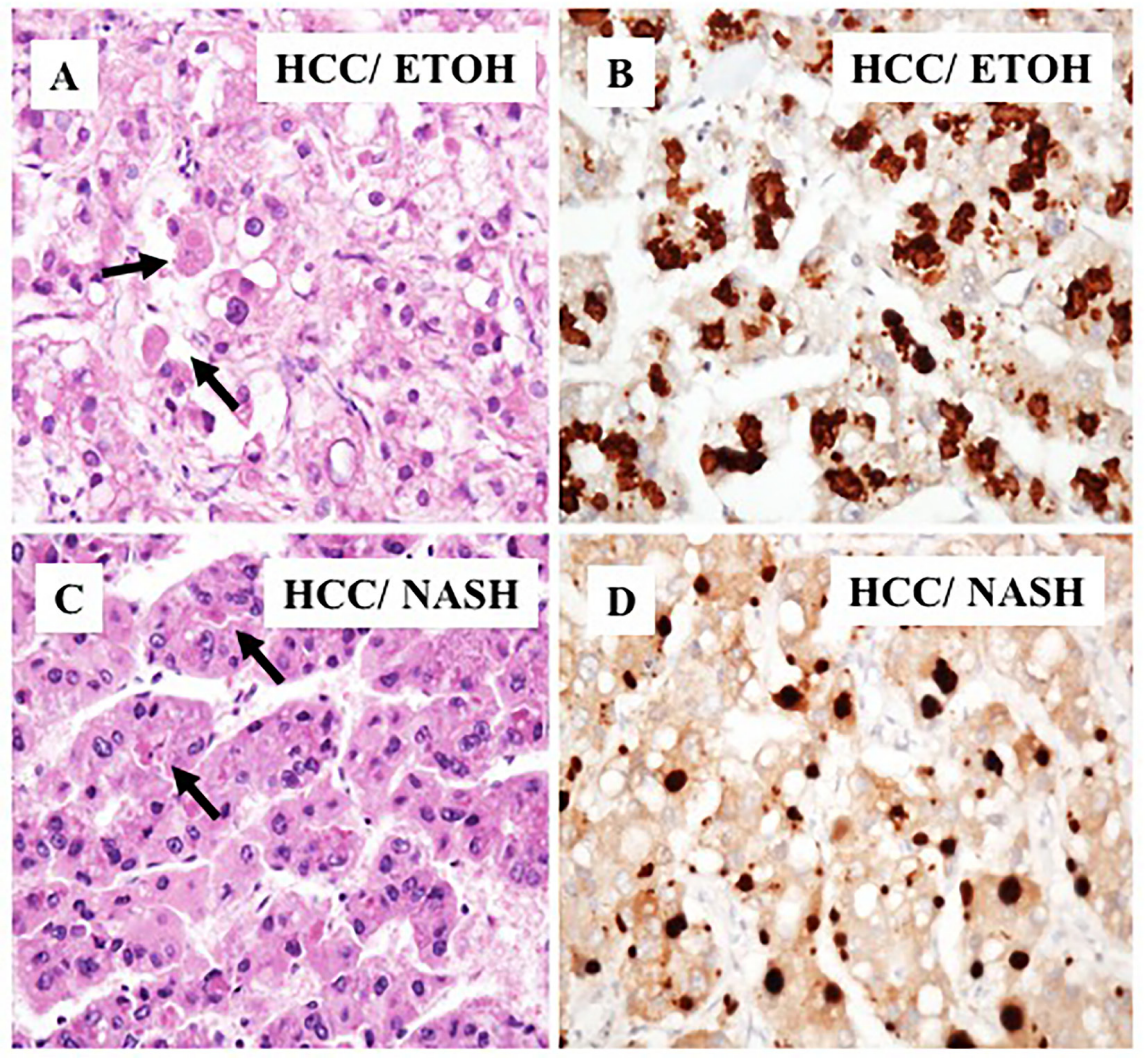
Immunohistochemical staining of HCC cells with Mallory-Denk bodies **A**. A representative samples of HCC present in alcoholic cirrhosis. Light microscopy of HCC that shows deposits of p62 in Mallory-Denk bodies. **B**. Immunostaining showing high deposition of p62 with Mallory-Denk bodies in tumor areas. **C**. Mallory-Denk bodies present in HCC related to NASH. **D**. Immunohistochemical staining of p62 deposition in Mallory-Denk bodies in HCC related to NASH.

Next, we compared p62 expression in HCCs and surrounding cirrhotic liver in samples of viral and non-viral etiologies and found similar levels of p62 expression between HCV-positive and HCV-negative HCCs. Strikingly, the number of p62-positive cells was significantly higher in HCCs compared to the adjacent cirrhotic liver tissue (*p* < 0.001). The number of p62-positive samples was highest in tissues from non-alcoholic steatohepatitis (NASH)-related HCCs (100%, 12/12), followed by 93% (15/16) in HCCs from HCV-related cirrhosis, 87% (7/8) in HCCs from alcoholic cirrhosis, and 70% (7/10) in HCCs from HBV-related cirrhosis (Figure 2, Supplementary Table 1). In a subset of the cases of non-viral etiology (NASH, alcoholic cirrhosis), we observed strong p62 staining associated with stress protein aggregates/deposits, called Mallory-Denk bodies (Figure 3). Mallory-Denk bodies (MDBs) and intracellular hyaline bodies (IHBs) are cytoplasmic inclusions found in a subset of HCCs. MDBs are mainly composed of intermediate filament protein keratin (K) 8 and K18, p62 and ubiquitin. We found presence of MDBs in 2 out of 8 (25%) alcoholic and 2 out 12 (16%) NASH related HCC, which is consistent with the study published by Ariane et. al. [29] showing that MDB are present in approximately 19% of HCC.

**Figure 4:**
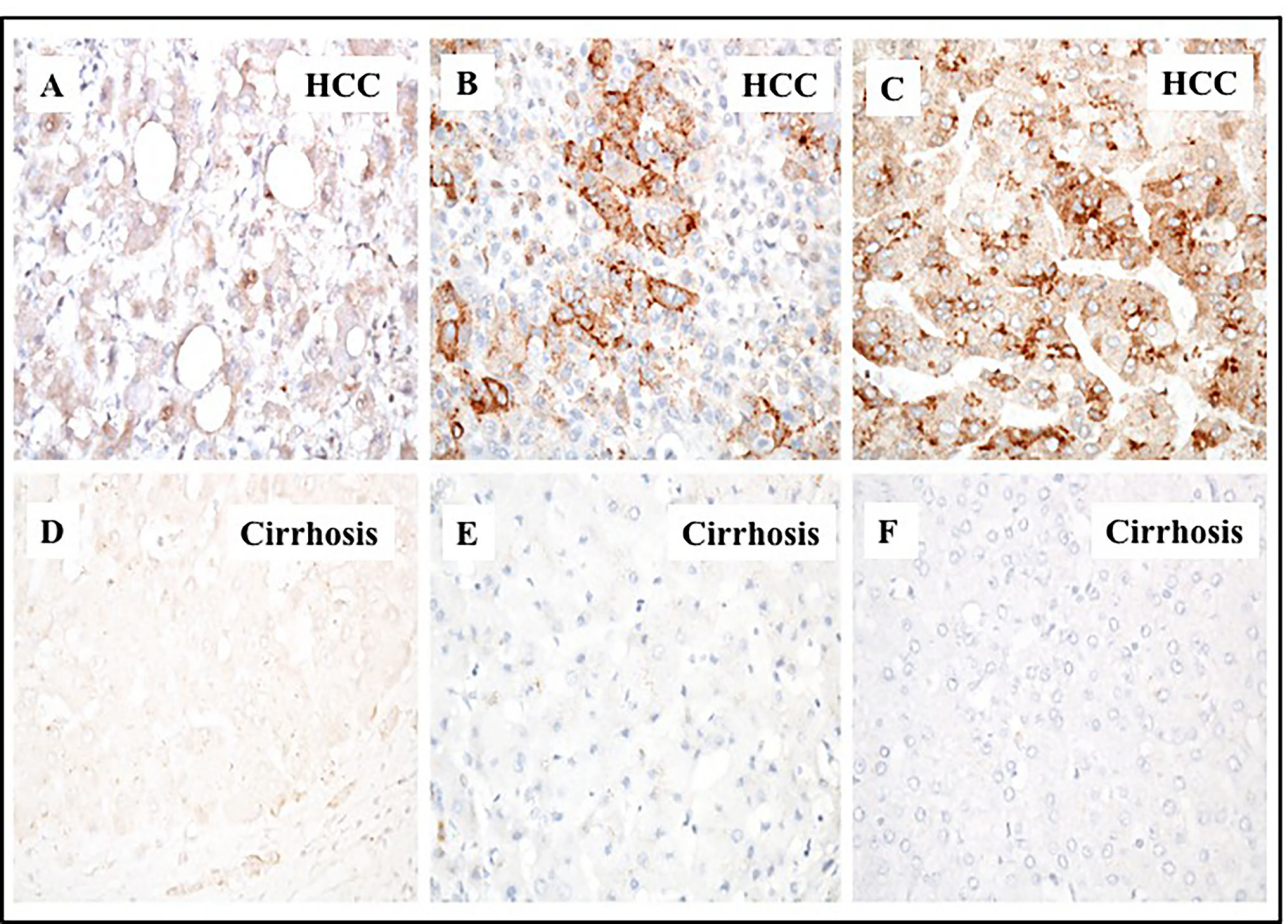
Immunohistochemical staining of glypican-3 protein in hepatocellular carcinoma and the adjacent non-tumorous cirrhotic liver A representative picture showing the proportion of glypican 3 staining immunopositive cells observed in cirrhotic livers with HCC.**A**. HCC with 1-10% cells are positive. **B**. HCC with 10-50% cells positive. **C**. HCC with 50-100% cell are positive for glypican-3 staining. **D**., **E**. and **F**. represents the negative staining in the adjacent non-tumorous tissues shown in corresponding panels **A**., **B**. and **C**. respectively. Pictures were taken at 40X magnification.

**Figure 5:**
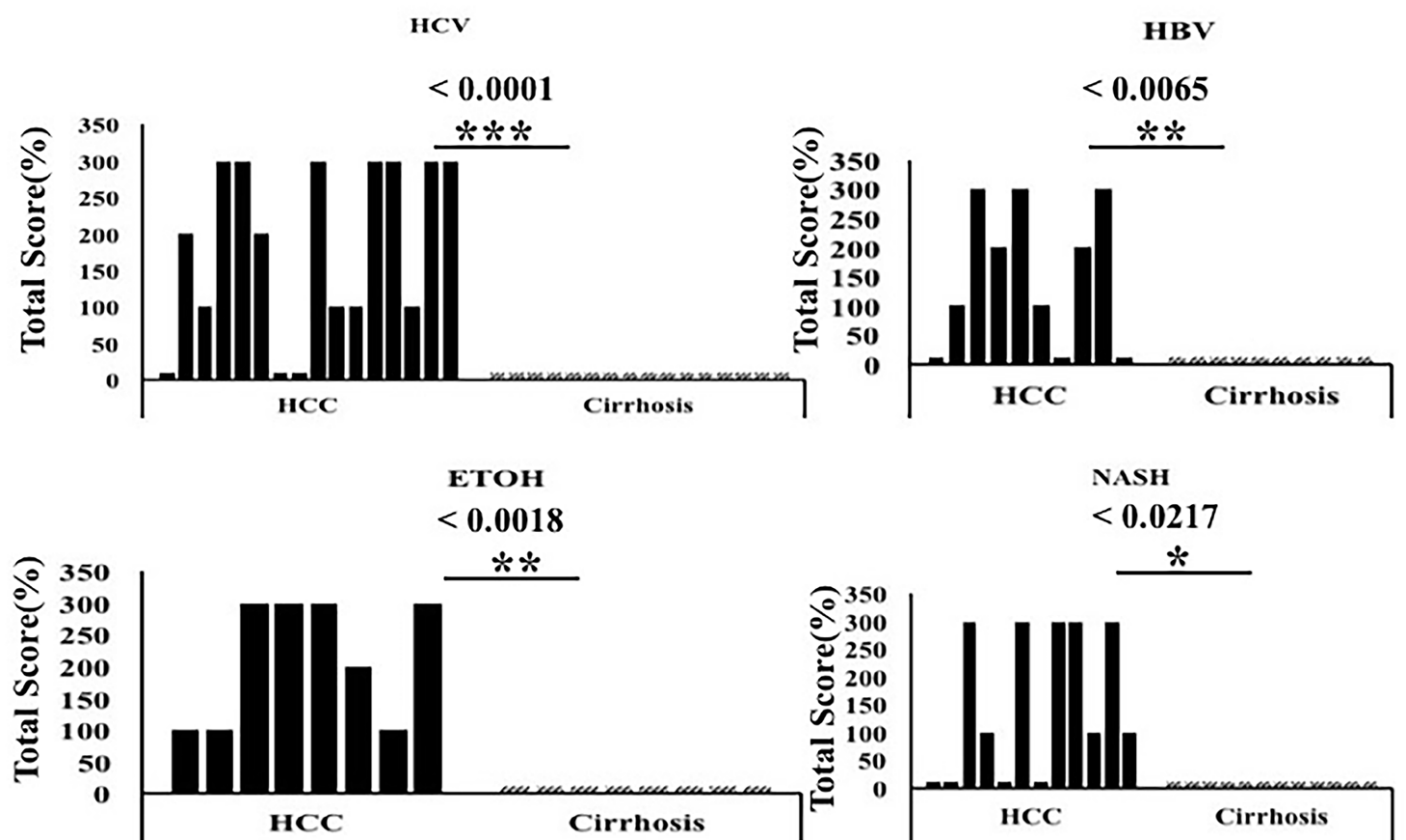
The expression of glypican-3 between HCC and non-tumorous cirrhotic liver tissues of different etiologies Staining intensity was semiquantified by considering the intensity of staining and the proportion of immunopositive cells. By multiplying the staining intensity score and the proportion of immunopositive cells, a staining score of 0-300 was determined. HCC cases showed increased glypican staining (median intensity 200; range 0-300), compared to the corresponding non-tumorous tissue of the cirrhotic liver (median 0, range 0-300). Glypican-3 staining was found to be significantly high in HCC of different etiologies as compared to the adjacent non-tumorous cirrhotic liver. Pictures were taken at 40X magnification. * *P* < 0.05, ** *P* < 0.001 and *** *P* < 0.0001.

**Figure 6:**
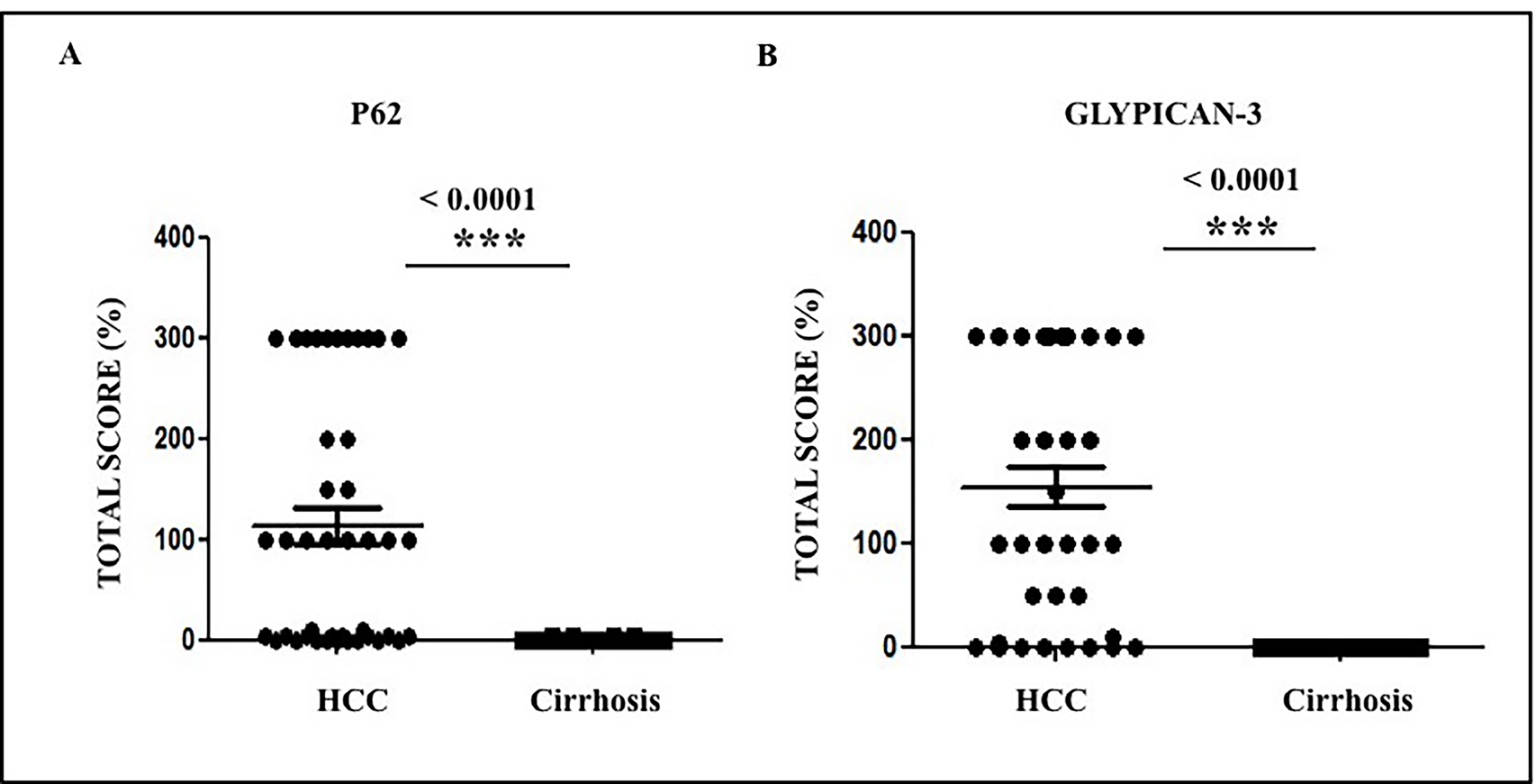
The expression of p62 and glypican-3 in tumor areas of 46 cirrhotic livers The expression of p62 and glypican-3 was significantly higher in HCC from viral and non-viral etiologies as compared to the adjacent non-tumorous cirrhotic liver. **A**. Staining score of p62 between HCC and cirrhosis. **B**. Staining score of glypican-3 between HCC and cirrhosis. * *P* < 0.05, ** *P* < 0.001 and *** *P* < 0.0001.

In a previous study, we showed that glypican-3 expression is induced in HCC due to an impaired macroautophagy response [26]. Glypican-3 (GPC3) belongs to the heparin sulfate proteoglycan family and promotes HCC growth by stimulating the WNT/β-catenin pathway [30]. In this study, we sought to determine if high levels of p62 correlated with glypican-3 expression. We found that 78% (36/46) of HCCs showed a variable degree of glypican-3 expression, while the adjacent cirrhotic liver tissue showed no staining for the protein. Samples from healthy livers also exhibited no glypican-3 expression (Figure 4). While the expression of p62 was mostly cytoplasmic, the expression of glypican-3 was both cytoplasmic and membranous. The number of glypican-3 positive cells was significantly higher in HCCs compared to adjacent cirrhotic liver tissue (*p* < 0.01). The number of glypican-3 positive cells was highest in alcohol-related HCCs (100%), followed by 81% (13/16) in HCV-related HCC, 70% (7/10) in HBV-related HCC and 66% (8/12) in HCC related to NASH (Figure 5, Supplementary Table 1). The number of glypican-3 positive cells was significantly higher in all HCCs when compared to the adjacent non-transformed cirrhotic livers (*p* < 0.001). Among the 39 samples that show p62 positive, 30 were glypican-3 positive and the p62 expression in HCC samples correlated well with glypican-3 expression; 84% and 78% respectively (Supplementary Table 1 and Supplementary Table 2). Among the ten glypican-3 negative HCC, eight showed positive expression for p62. Two samples were negative for both the markers. Taken together, the median intensity of expression of p62 and glypican-3 was significantly higher in HCCs compared to the adjacent non-transformed hepatocytes in the cirrhotic liver (Figure 6, *p* < 0.01). No statistically significant differences were observed in the expression of p62 and glypican-3 protein between HCC etiologies.

### LAMP-2A expression is increased in HCC and adjacent non-tumorous cirrhotic liver

**Figure 7:**
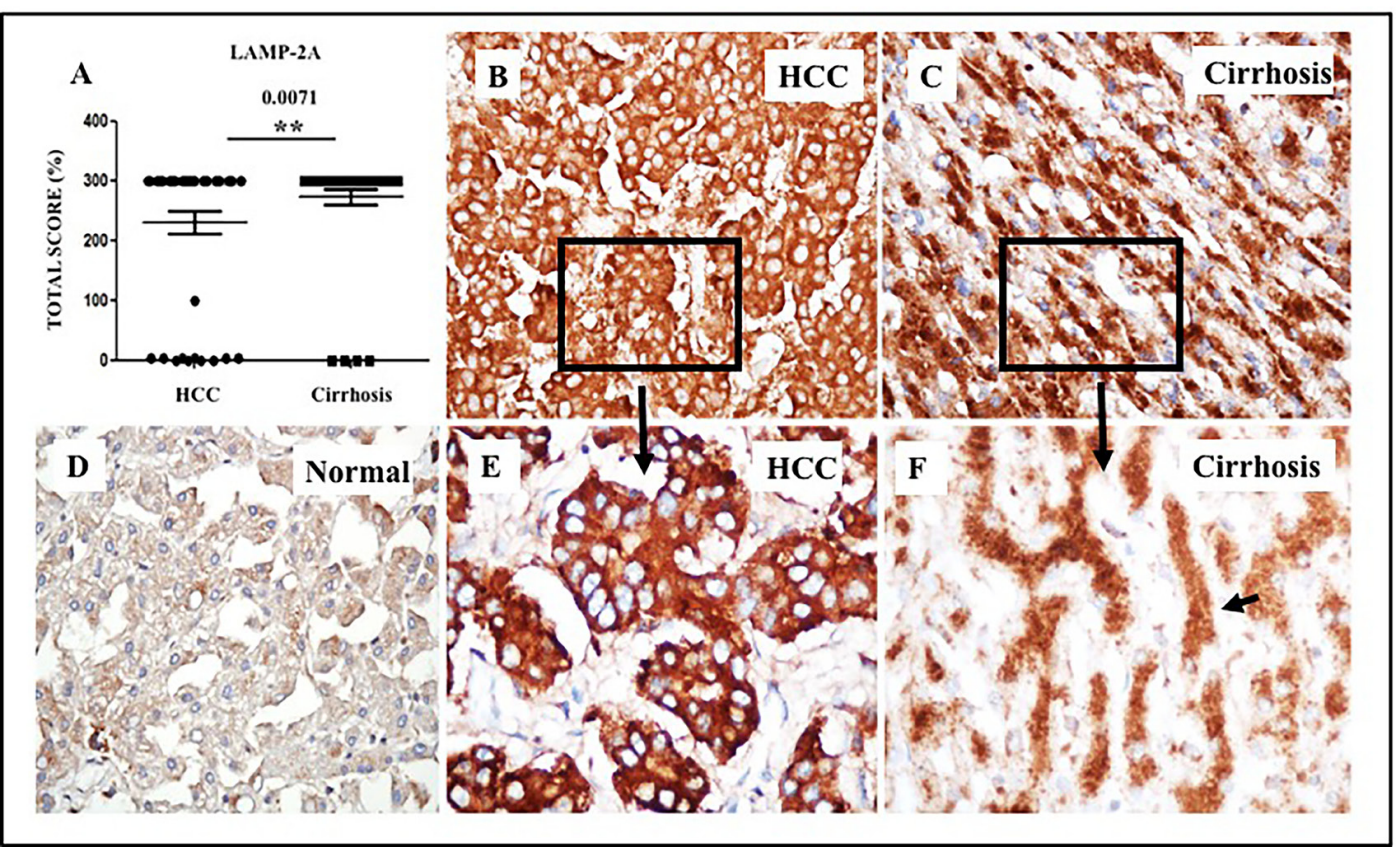
Expression of LAMP-2A protein in HCC and corresponding non-tumorous liver tissue Staining intensity was semiquantified by considering the intensity of cytoplasmic staining and the proportion of immunopositive cells. A staining score of 0-300 was determined by multiplying the staining intensity score and the proportion of immunopositive cells. **A**. Median staining score of LAMP-2A between HCC and adjacent non-tumorous cirrhotic liver. **B**. A representative staining of LAMP-2A in HCC. LAMP-2A expression is altered in HCC. **C**. A representative staining of LAMP-2A in non-tumorous cirrhotic liver. LAMP-2A staining in non-tumorous hepatocytes of the cirrhotic livers show cytoplasmic and membranous staining pattern with accentuation adjacent to bile canalicular compartment. **D**. A representative picture of LAMP-2A staining of normal liver. Pictures were taken at 40X magnification. * *P* < 0.05, ** *P* < 0.001 and *** *P* < 0.0001. **E**. Canalicular accentuation of LAMP-2A staining is absent in HCC. Most of the LAMP-2A staining is cytoplasmic. **F**. High magnification (60x) picture shows canalicular accentuation of LAMP-2A in cirrhotic liver.

The only reliable marker used for assaying CMA is the lysosomal receptor LAMP-2A. Substrate binding to LAMP-2A is an essential step for CMA-mediated protein degradation in the lysosome. Cells modulate expression of LAMP-2A to increase or decrease CMA activity [23, 31]. We examined the expression of LAMP-2A between HCC and cirrhotic nodules to determine whether induction of CMA could contribute to HCC survival in cirrhotic liver. We performed semiquantitative immunohistochemical analysis of LAMP-2A expression in paraffin-embedded tissue sections from HCCs and surrounding non-transformed, cirrhotic liver tissue. We detected LAMP-2A expression in 95% (44/46) of HCCs from cirrhotic livers (Supplementary Table 1, Figure 7). LAMP-2A expression was also high in non-transformed hepatocytes present in the cirrhotic livers. The expression of LAMP-2A was low in 8 normal tissues without liver cirrhosis, compared to HCCs examined under similar conditions (Figure 7D). Those two HCC samples, which were negative for LAMP-2A staining, showed positive staining for p62 and glypican-3.

Strikingly, we observed that the LAMP-2A staining pattern is different in HCCs, compared to surrounding non-transformed liver (Figure 7C). In non-transformed cirrhotic liver cells, LAMP-2A localization was both cytoplasmic and membranous, with intense staining adjacent to the bile canalicular compartment (Figure 7F). However, the canalicular compartmentalization of LAMP-2A staining was frequently lost in HCC (Figure 7E). In some HCC, the LAMP-2A staining was heterogenous, either patchy or negative, while well-differentiated HCCs maintained LAMP-2A staining compartmentalization. In some HCC, there was a gradual alteration in the LAMP-2A staining pattern when transitioning from the cirrhotic region to HCC.

**Figure 8:**
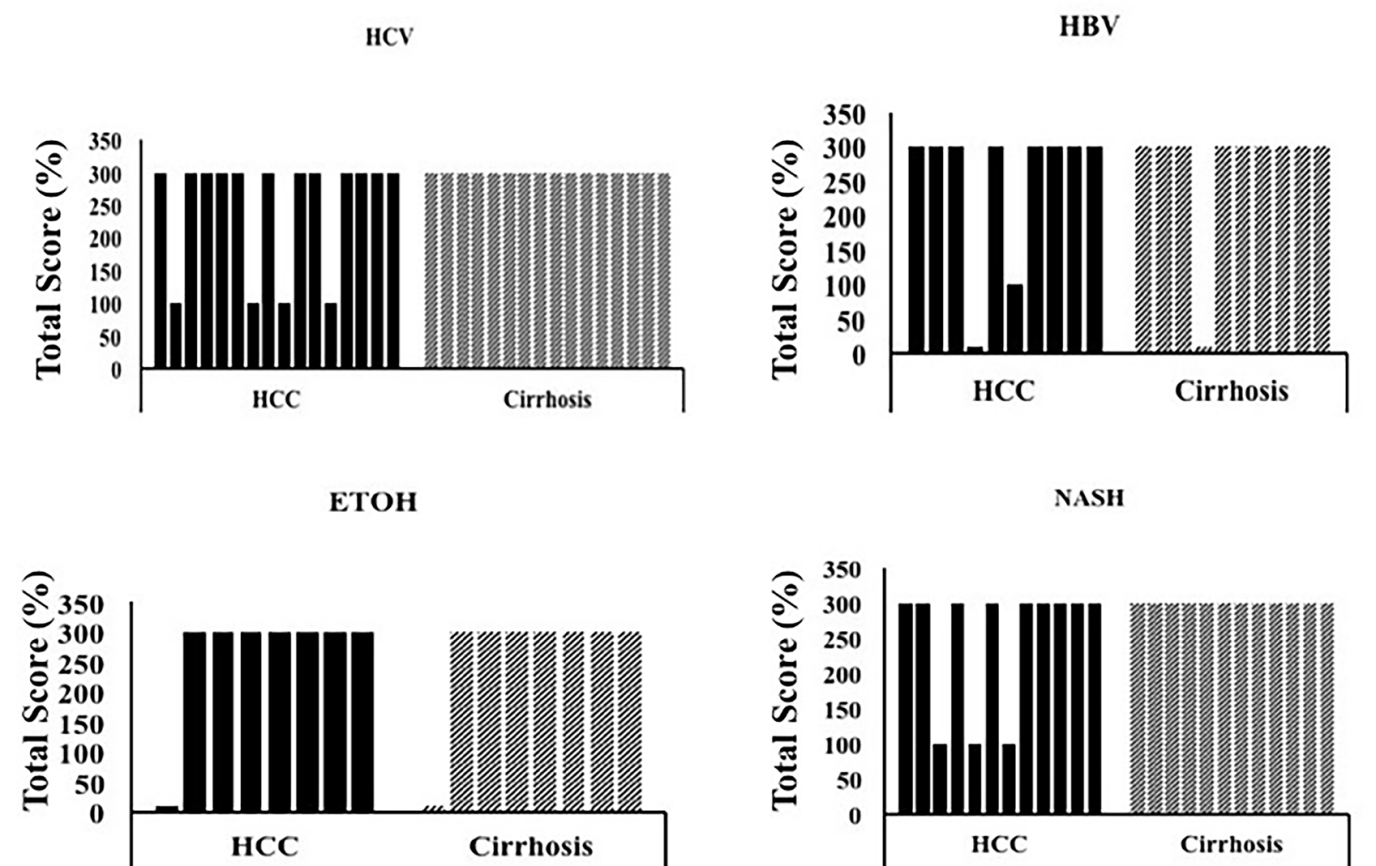
LAMP-2A expression between HCC and non-tumorous cirrhotic liver tissues of different etiologies Staining was semiquantified by considering the intensity of staining and the proportion of immunopositive cells. A staining score of 0-300 was determined by multiplying the staining intensity and the proportion of immunopositive cells. LAMP-2A staining was found to be high in HCC and adjacent non-tumorous cirrhotic liver. * *P* < 0.05, ** *P* < 0.001 and *** *P* < 0.0001.

We next checked whether LAMP-2A expression varied between HCCs related to viral and non-viral etiologies. The expression of LAMP-2A was present in both HCC and surrounding non-transformed cirrhotic liver tissue in samples of viral (HCV and HBV) and non-viral etiology (alcoholic and NASH) (Figure 8). Overall, the LAMP-2A localization was significantly altered in the HCC regions of all samples compared to the non-transformed regions. There were no significant differences in the LAMP-2A staining patterns between viral and non-viral HCCs and surrounding non-transformed cirrhotic livers (Supplementary Table 3).

### Decreased macroautophagy flux correlates with activation of CMA in HCC of viral and non-viral etiologies

**Figure 9:**
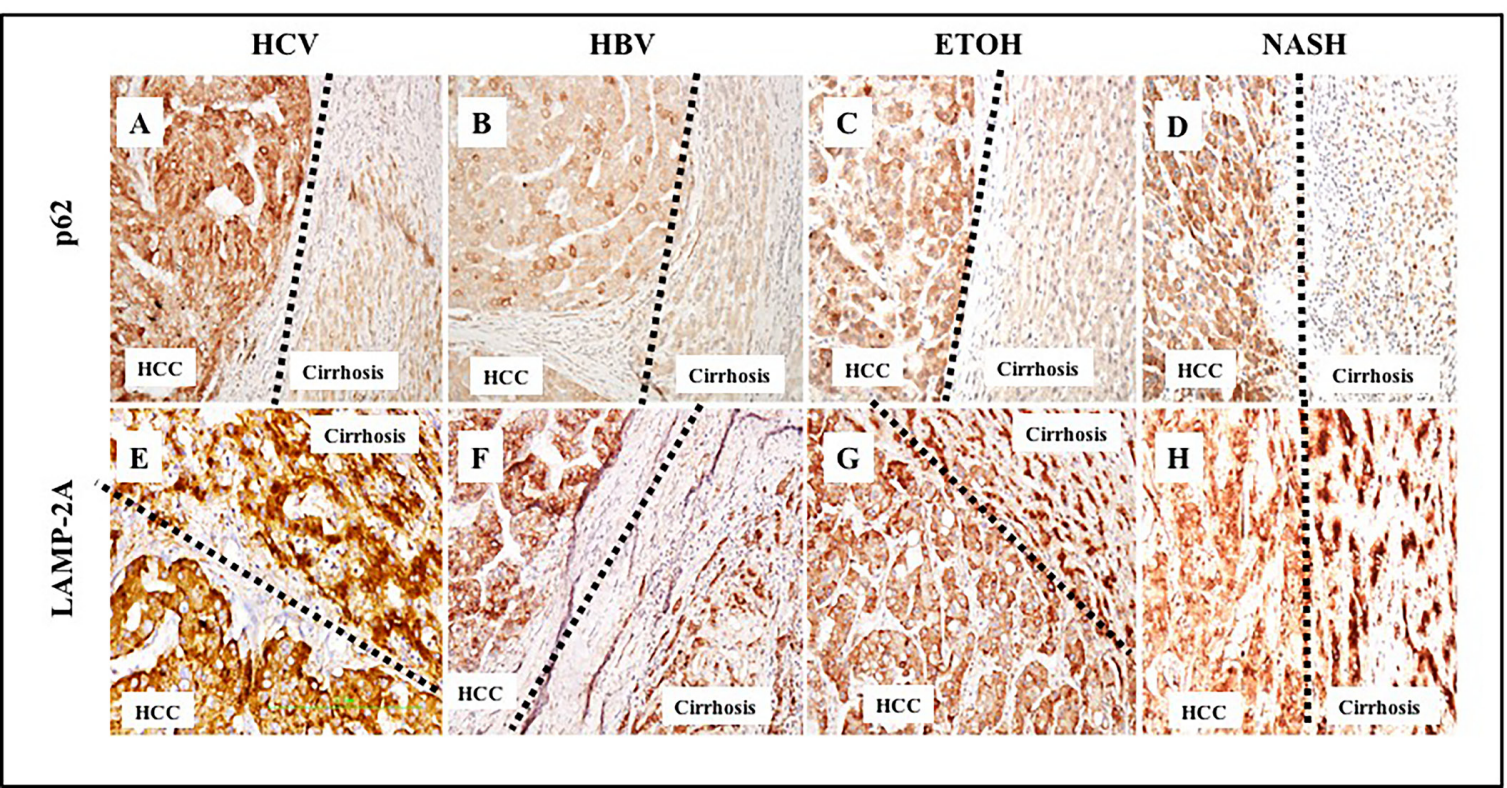
Comparative assessment of macroautophagy (inversely correlates with p62 expression) and chaperone-mediated autophagy (LAMP-2A expression directly correlates with CMA) among 46 cirrhotic liver samples with HCC **A**. & **E**. HCV infected liver cirrhosis with HCC. HCC are p62 positive (impaired macroautophagy) and altered expression of LAMP-2A (high CMA). Non-tumorous cirrhotic livers are p62 negative (active macroautophagy) and LAMP-2A induced but not degraded in cirrhosis (impaired CMA). **B**. & **F**. HBV infected liver cirrhosis with HCC. HCC are p62 positive (impaired autophagy) and altered expression of LAMP-2A (high CMA). Non-tumorous cirrhotic livers are p62 negative (active macroautophagy) and LAMP-2A was induced but not degraded in cirrhosis (impaired CMA). **C**. & **G**. Alcohol related liver cirrhosis with HCC. HCC are p62 positive (impaired macroautophagy) and altered expression of LAMP-2A (high CMA). Non-tumorous cirrhotic livers are p62 negative (active macroautophagy) and LAMP-2A induced but not degraded in cirrhosis (impaired CMA). **D**. and **H**. NASH related liver cirrhosis with HCC. HCC are p62 positive (impaired macroautophagy) and altered expression of LAMP-2A (high CMA). Non-tumorous cirrhotic livers are p62 negative (active macroautophagy) and LAMP-2A was induced but not degraded in cirrhosis (impaired CMA). Pictures were taken at 20X magnification.

We performed a comparative analysis to study the inverse relationship between macroautophagy (associated with lack of p62 expression) and CMA (associated with LAMP-2A expression) in HCC and the adjacent non-transformed cirrhotic liver. Cancer nodules from poorly differentiated carcinoma showed a uniformly strong expression of p62 compared to the surrounding non-malignant hepatocytes. The expression of p62 was mostly cytoplasmic in HCCs, consistent with the p62 localization observed in hepatoma cell lines grown in culture [26]. The pattern of p62 expression between the cirrhotic area and the tumor was compared in 46 specimens comprising various etiologies (HBV, HCV, alcohol, and NASH). We consistently observed uniformly high expression of p62 in HCC and negative p62 expression in the cirrhotic liver (Figure 9A-9D, upper panel). LAMP-2A localization was also strikingly different between cirrhotic non-transformed hepatocytes and HCCs, regardless of cirrhotic etiology (Figure 9E-9H, lower panel). The presence of increased LAMP-2A expression in cirrhotic liver and its altered localization in the tumor areas suggest that CMA may be involved in HCC survival.

### CMA compensates for defective macroautophagy in cultured HCC cells

**Figure 10:**
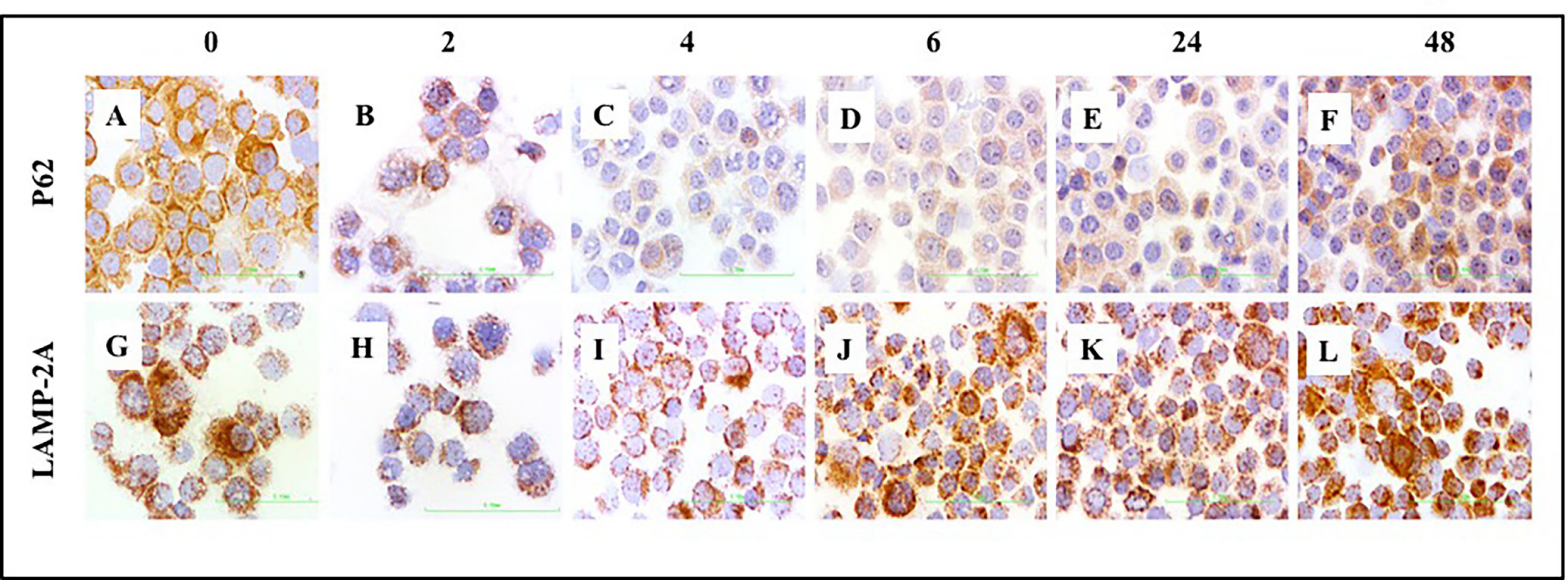
Macroautophagy and CMA-related protein expressions in serum starved Huh-7.5 cells Huh-7.5 cells were cultured in serum free medium for indicated time points. The expression of p62 (upper panel) and LAMP-2A protein was measured by immunostaining of cytospin slides using Vectastain kit. **A**.-**F**. Decreased expression of p62 at 2, 4, and 6 hours represents activation of macroautophagy and increased expression of p62 at 24 and 48 hours means macroautophagy is impaired. G-L. Induced expression of LAMP-2A at 6, 24 and 48 hours suggest that CMA is activated when macroaputophagy is impaired. HCC are p62 positive (impaired macroautophagy) and altered expression of LAMP-2A (high CMA). Non-tumorous cirrhotic livers are p62 negative (active macroautophagy) and LAMP-2A induced but not degraded in cirrhosis (impaired CMA). (Picture are taken at 40X magnification).

To understand the interaction between macroautophagy and CMA activation, we analyzed expression of p62 and LAMP-2A by immunohistochemistry in Huh-7.5 cells cultured with serum-free media for 0, 2, 4, 6, 24, and 48 hours. Consistent with previously published results [24, 25], we found that activation of macroautophagy correlates with decreased expression of p62 in serum-starved Huh-7.5 cells after 2, 4, and 6 hours (Figure 10A-10F, top panel). To further understand the relationship between macroautophagy and CMA in serum-starved cells, we examined expression of LAMP-2A by immunocytochemical staining. LAMP-2A expression increased after 24 and 48 hours of serum starvation (Figure 10G-10L, bottom panel). Similarly, expression of p62 was increased at 24 and 48 hours, consistent with impaired macroautophagy flux. Our results thus far suggest that macroautophagy and CMA were not induced at the same time and that CMA was induced after 24 and 48 hours, concomitant with a decrease in macroautophagy flux in serum-starved Huh-7.5 cells.

### Expression of glucose-regulated protein 78 (GRP78) and heat shock cognate protein (Hsc70) is elevated in liver cirrhosis and HCC

**Figure 11:**
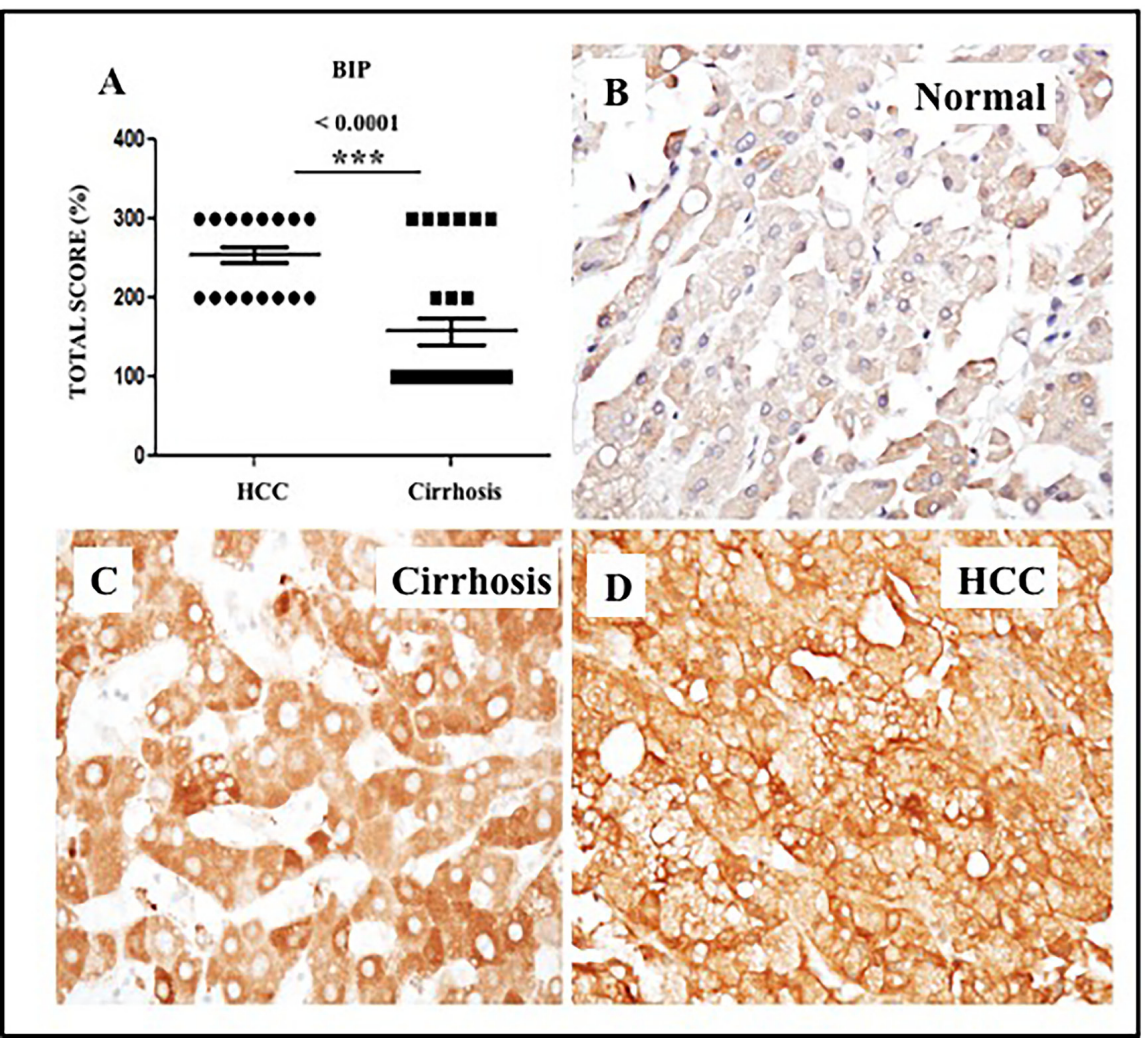
Expression of ER-stress chaperone (GRP78) proteins in HCC and adjacent non-tumorous cirrhotic liver **A**. Showed a trend for higher expression of GRP78 in HCC as compared to non-tumorous cirrhotic liver. Staining was semiquantified by considering the intensity of staining and the proportion of immunopositive cells. By multiplying the staining intensity score and the proportion of immunopositive cells a staining score of 0-300 was determined. **B**. Representative image showing the expression of GRP78 in normal liver. **C**. Representative image of GRP78 protein expression in liver cirrhosis. **D**. Representative image of GRP78 protein staining in HCC. (Original magnification X40). * *P* < 0.05, ** *P* < 0.001 and *** *P* < 0.0001.

We have previously shown that endoplasmic reticulum stress (ER stress) and the unfolded protein response (UPR) are activated in cell culture as a result of HCV infection, alcohol, and treatment with free fatty acids [32, 33]. The 78-kD glucose-regulated protein GRP78 (BiP) is a molecular chaperone induced in response to ER stress, as well as chronic liver disease and liver cirrhosis [32]. We sought to define the role of ER stress in HCCs and the surrounding cirrhotic tissues by measuring the expression of GRP78 by semiquantitative immunohistochemical analysis of tissue sections from 26 cirrhotic livers with HCC. Among the 26 cirrhotic livers with HCC, 100% showed increased expression of GRP78 in tumor areas compared to the non-transformed hepatocytes in the adjacent cirrhotic liver (Figure 11), suggesting that ER stress persists during the progression of HCC in the cirrhotic liver. In contrast, normal liver samples without cirrhosis showed only a small amount of GRP78 staining in the cytoplasm (Figure 11B). We also found that GRP78 expression was significantly increased in liver cirrhosis tissues and HCCs in the samples of viral and non-viral etiologies (Supplementary Table 4).

**Figure 12:**
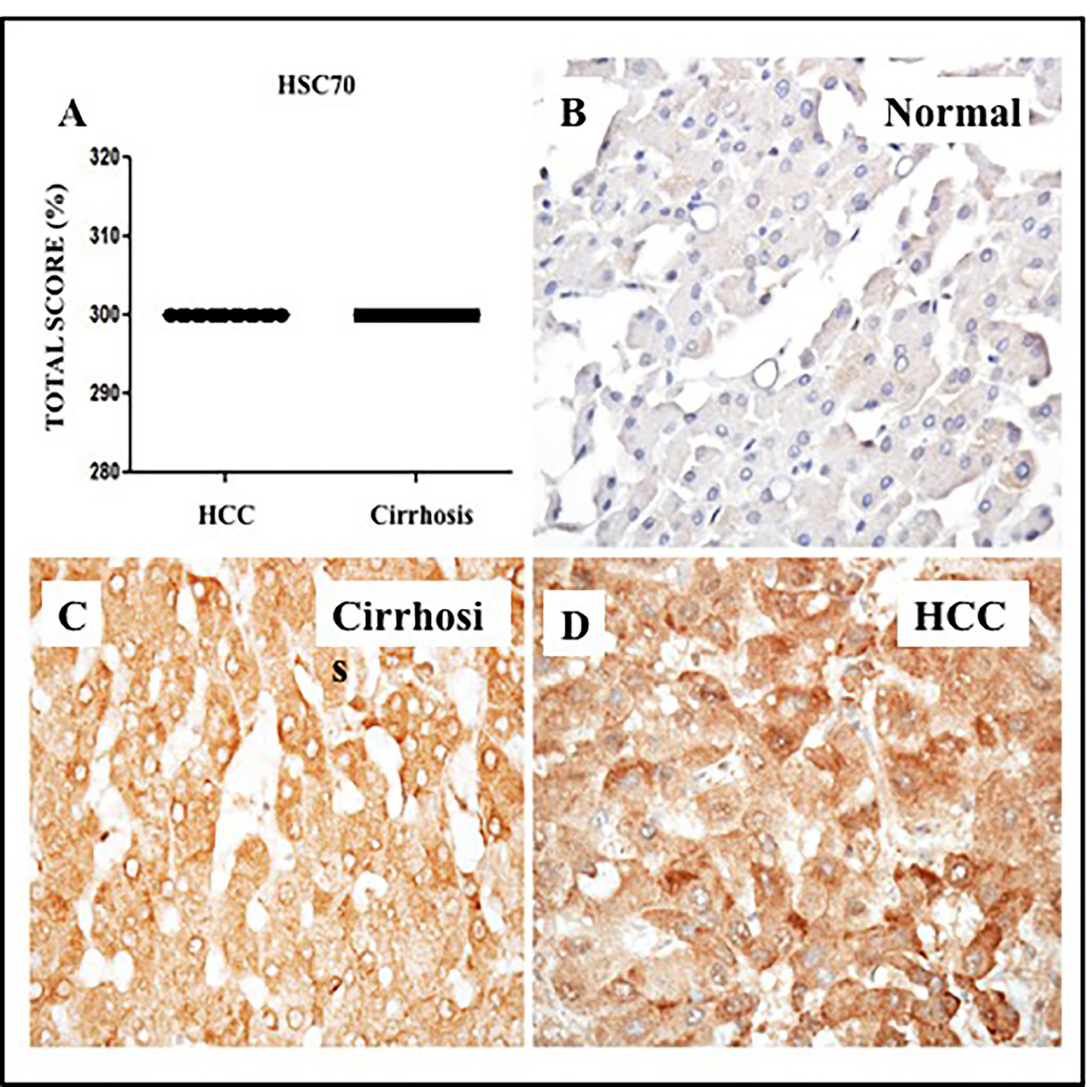
Expression of heat shock cognate protein (Hsc70) in HCC and adjacent non-tumorous cirrhotic liver **A**. Showed a trend for higher expression of GRP78 in HCC and non-tumorous cirrhotic liver. Staining was semiquantified by considering the intensity of staining and the proportion of immunopositive cells. By multiplying the staining intensity score and the proportion of immunopositive cells a staining score of 0-300 was determined. **B**. Representative image showing the expression of Hsc70 in normal liver. **C**. Representative image of Hsc70 protein expression in liver cirrhosis. **D**. Representative image of Hsc70 protein staining in HCC. (Original magnification X40). * *P* < 0.05, ** *P* < 0.001 and *** *P* < 0.0001.

Given that Hsc70 is a chaperone necessary for lysosomal degradation of cytosolic proteins during CMA, we examined expression of Hsc70 in HCCs and non-tumorous cirrhotic livers by immunohistochemical analysis. Of the 26 cirrhotic livers with HCC, 100% of HCCs and the surrounding non-tumorous cirrhotic livers displayed high Hsc70 expression (Figure 12A). Low-level expression of Hsc70 was present in normal livers (Figure 12B). In contrast, Hsc70 expression was high in the cirrhotic liver (Figure 12C) and HCC samples as well (Figure 12D), consistent with induction of stress chaperones (BiP and Hsc70) in liver cirrhosis and HCC (Supplementary Table 4). Expression of ER stress chaperones (GRP78 and Hsc70) correlated with that of p62, glypican-3 and LAMP-2A (Supplementary Figure 1). Since we used a limited number of HCC samples in this investigation, the immunostaining data were correlated with patients prognosis using 442 HCC cohort available on TCGA database, freely available online. We found all the markers show induced mRNA expression in this HCC cohort. Kaplan-Meier survival analysis of TCGA datasets, consisting of 442 HCC patients, show that SQSTM1/p62 and HSC70 expression significantly correlated with worse survival rates (Supplementary Figure 2). Our data are also supported by a recent publication by Michael Karin's group that suggests that p62 accumulation in the non-tumor liver tissues of early-stage HCC patients undergoing curative ablation is associated with HCC recurrence and relapse. They found that the disease free survival rate was much lower in patients with high p62 expression in non-tumor liver than in patients with low or no p62 staining [34].

### Autophagy inhibition decreases HCC proliferation and induces cell death

**Figure 13:**
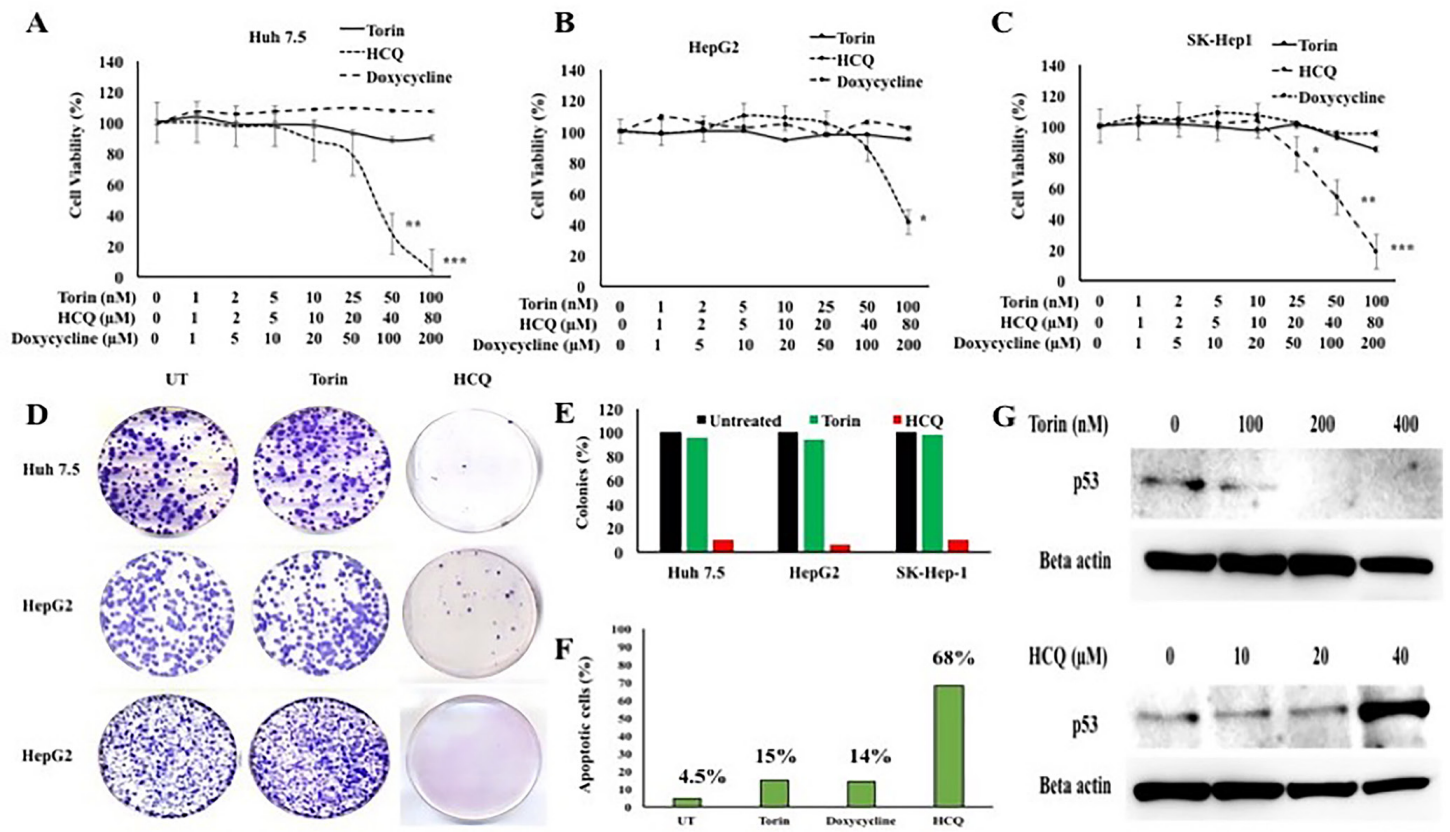
HCC cell proliferation in the presence of autophagy inducer, Torin 1 and autophagy inhibitor, HCQ Three different HCC cell lines (Huh-7.5, HepG2 and SK-Hep-1) were seeded in 24 well tissue culture plates and then treated with increasing concentrations of either Torin 1 or HCQ. MTT assay was performed after 72 hours. **A**. Proliferation of Huh7.5 cells in the presence of Torin 1 or HCQ. **B**. Proliferation of HepG2 cells in the presence of Torin 1 and HCQ. **C**. Proliferation of SK-Hep-1 in the presence of Torin 1 and HCQ. **D**. Cell colony assay showing the long-term proliferation of three HCC cell lines treated with autophagy inducer (Torin1) or lysosomal inhibitor (HCQ). **E**. Quantitation of cell colonies that survived drug treatment. **F**. Huh-7.5 cells were seeded at 1×10^5^ per ml in 6 well tissue culture dish. The next day, cells were treated with Torin (100nM), HCQ (80μM) or doxycycline (200μM) for 48 hours. After this step, cells were isolated by trypsin-EDTA and washed with PBS then incubated with indicated concentartions of FITC-annexin V and propodium iodide (PI) (BD Biosciences) in binding buffer for 15 minutes in dark. Stained cells were immediately subjected to flow cytometry analysis using FACS Calibur flow cytometer (BD Biosciences, San Diego, CA). Flow cytometric evaluation of apoptosis after treatment with Torin 1, or HCQ or Doxycycline. **G**. Western blot shows the expression of p53 tumor suppressor in Huh7.5 cells treated with increasing concentration of autophagy inducer Torin 1 and autophagy inhibitor HCQ.

To determine whether inhibition of macroautophagy flux or the presence of CMA promotes HCC survival in the cirrhotic liver, we measured HCC proliferation and long-term survival in culture in the presence of an autophagy inducer or an autophagy inhibitor. A recent study shows that TORC1 inhibition induces macroautophagy whereas TORC2 inhibition activates CMA [35]. The authors showed that increasing concentration of Torin1 treatment resulted in a dose-dependent increase in CMA activity, whereas rapamycin that only inhibits TORC1 did not change CMA activity. These results suggest that Torin1 induced CMA by inhibition of TORC2 activity. In our study, three different HCC cell lines (Huh-7.5, HepG2, and SK-Hep 1) were treated with an increasing concentration of Torin1 or hydroxychloroquine (HCQ, a lysosomal inhibitor). We then measured cell proliferation by MTT assay after 72 hours. As shown in Figure 13A-13C, HCQ treatment decreased proliferation significantly in all three HCC cell lines, but autophagy induction by Torin 1 or treatment with a control drug, doxycycline, did not inhibit HCC proliferation. Long-term proliferation of Huh-7.5 cell line after treatment with HCQ or Torin 1 was determined by colony formation assay (Figure 13D). The number of cell colonies that survived due to long-term treatment with HCQ or Torin-1 was examined by Image J software and demonstrated that HCQ inhibits HCC proliferation and inhibits HCC growth in long-term cultures, whereas Torin 1 treatment promotes HCC survival (Figure 13E). A flow cytometry based annexin V and propidium iodide (PI) staining assay was used to quantitate apoptotic Huh-7.5 cells treated with Torin 1, HCQ and Doxycycline (Supplementary Figure 3). Clearly, HCQ treatment at 48 hours induced 68% apoptosis in Huh-7.5 cells as compared to minimal cell death due to Torin 1 treatment (Figure 13F). Two different studies have shown that p53 tumor suppressor is a CMA substrate protein that is degraded in lysosome dependent manner [36,37]. We examined the mechanism underlying HCQ-mediated decreases in HCC proliferation by analyzing expression of p53 levels by Western blot. Results shown in Figure 13G indicate that HCC cultures treated with HCQ induced p53 expression, whereas Torin1 treatment promoted p53 degradation by CMA. These results support the hypothesis that inhibition of autophagy decreases cell proliferation and increases cell death through induction of the p53 tumor suppressor. Taken together, our data show that impaired macroautophagy is balanced by activation of CMA in HCC, suggesting that an autophagy compensatory mechanisms are involved in HCC survival in the cirrhotic liver.

## DISCUSSION

The risk of HCC development is increased significantly among the patients with advanced liver fibrosis or cirrhosis related to viral and non-viral etiologies. Although it is well known that HCC develops more frequently in the background of liver cirrhosis, the molecular mechanisms underlying the progression of liver cirrhosis to HCC remain unclear.

The endoplasmic reticulum stress (ER stress) and the unfolded protein response (UPR) persist during chronic liver disease and liver cirrhosis related to viral infection (HCV, HBV) and non-viral etiologies (alcoholic and non-alcoholic steatohepatitis, NASH). We showed previously that expression of UPR genes (BiP, peIF2α, IRE1α) are induced in liver biopsies of chronic HCV patients and stage IV fibrosis (cirrhotic livers) [32]. Shuda, et al. [38] showed expression of ATF6, XBP1 and GRP78 were induced in HCC, suggesting that ER-stress pathway may be involved in HCC development. This study showed that ER stress is unequivocally high in all HCC as well as surrounding non-tumorous cirrhotic liver. The presence of ER-stress/UPR activation has been found in chronic liver diseases related to hepatitis B virus (HBV) infection. Accumulation of mutant forms of hepatitis B surface antigen (HBsAg) in the infected hepatocytes that leads to expansion of ER and development of ground glass hepatocytes and hepatocellular carcinoma development is another example of viral carcinogenesis associated with ER-stress [39]. A recent review article nicely describes how ER stress plays a central role in the progression of alcoholic and non-alcoholic fatty liver diseases [40]. A number of recent publications demonstrate the importance of ER-stress association with subsequent development of liver cirrhosis and HCC [41–44]. These findings are also supported by a number of excellent reviews summarizing the impact of ER-stress and UPR activation in the evolution of chronic liver disease and cancer [45,46]. However, the molecular mechanism for how hepatic UPR response links to HCC development is not completely understood.

Hepatic ER stress triggers the autophagic degradation process to promote hepatocyte survival during viral infection and non-viral insults [19]. Autophagy is a lysosomal degradation process needed for energy balance and cell survival under different stress conditions, including viral infection, nutrient deprivation, hypoxia, and ischemia. Autophagy plays a major role in the pathogenic mechanisms of liver disease during progression from chronic liver disease to cirrhosis, and finally to hepatocellular carcinoma [47,48]. Several reviews have summarized the work from different laboratories that addresses the role of ER stress and autophagy in the progression of alcoholic and non-alcoholic fatty liver diseases [49–51]. In general, autophagy may play a role in tumor suppression through the maintenance of cellular homeostasis during chronic stages of liver disease and cirrhosis, which are associated with increased ER stress for both viral and non-viral etiologies. The crosstalk between autophagy and cell death (apoptosis and necrosis) in chronic liver disease balances tumor suppression and tumor progression [51].

Recent studies found that mice harboring liver-specific deletions of ATG5 or ATG7 or heterozygous deletion of Beclin, develop hepatic neoplasia more frequently than wild-type mice [52,53], suggesting that autophagy inhibition is associated with HCC development. To understand whether similar mechanisms can be seen in human HCC, we found that most HCCs present in cirrhotic liver show increased expression of p62, suggesting that insufficient macroautophagy exists in most HCCs, as compared to the non-tumorous cirrhotic liver [26]. Consistent with these observations, Ding, et. al. [27] showed that decreased expression of autophagy genes and autophagy activities correlates with more aggressive cancer and poor prognosis of HCC. These findings are in agreement with prior studies in mice that showed that defects in macroautophagy resulting from heterozygous or homozygous deletion of autophagy genes accelerate hepatocarcinogenesis. The mechanisms by which cancer cells survive with a defective macroautophagy response are unknown.

In the current study, we have extended our observations by examining whether impaired macroautophagy is also present in HCCs derived from viral and non-viral etiologies. We found that most of the cirrhotic livers are p62 negative, suggesting that the autophagy flux protein is degraded in the cirrhotic livers due to increased macroautophagy response. We find most of the HCC areas show accumulation of autophagy flux protein p62. Since p62 itself is an autophagy substrate, accumulation of p62-containing protein aggregates is regarded as impaired macroautophagy response in HCC. According to a previous report [24] and our serum starvation experiments of cultured cells, CMA only works when the macroautophagy becomes defective. Macroautophagy and CMA do not work simultaneously. They can compensate for each other. This is supported by immunostaining data of LAMP-2A between HCC and the surrounding non-tumorous cirrhotic liver. The LAMP-2A expression and subcellular distribution was found to be dramatically different in tumor *versus* cirrhotic areas across all samples examined. Most of the cirrhotic areas and normal non-cirrhotic livers show LAMP-2A staining that is compartmentalized to the pericanalicular membranous areas of the cell, whereas in tumors, staining was mostly diffuse cytoplasmic. Based on these analogies, we believe that CMA is activated only in the HCC, not in the cirrhotic livers. However, our immunostaining based detection of autophagy protein expression cannot exclude the possibility of low-basal level activation of CMA due to impaired macroautophagy at the cirrhosis stage. The exact stage when the autophagy switch occurs in the cirrhotic liver during the HCC development needs to be verified experimentally.

We also observed heterogeneous expression of LAMP-2A, with high LAMP-2A in some areas of the neoplasm and low LAMP-2A expression in other areas. Several factors may explain why LAMP-2A expression is heterogeneous within the tumors [54–56]. (i) Lysosomal storage disorders may alter LAMP-2A expression in the lysosome membrane. (ii) Nutrient deprivation could activate macroautophagy in HCC, which could decrease CMA through LAMP-2A degradation. (iii) LAMP-2A stability could be decreased due to proteolytic cleavage of LAMP-2A at the lysosomal membrane by lysosomal proteases. (iv) LAMP-2A concentrations could be altered by changes in the dynamic distribution of LAMP-2A between the lysosomal membrane and matrix. (v) Accumulation of dietary lipids in tumor cells could inhibit LAMP-2A expression. (vi). Lysosomal activation of mTORC2-Akt pathway [35].

**Figure 14:**
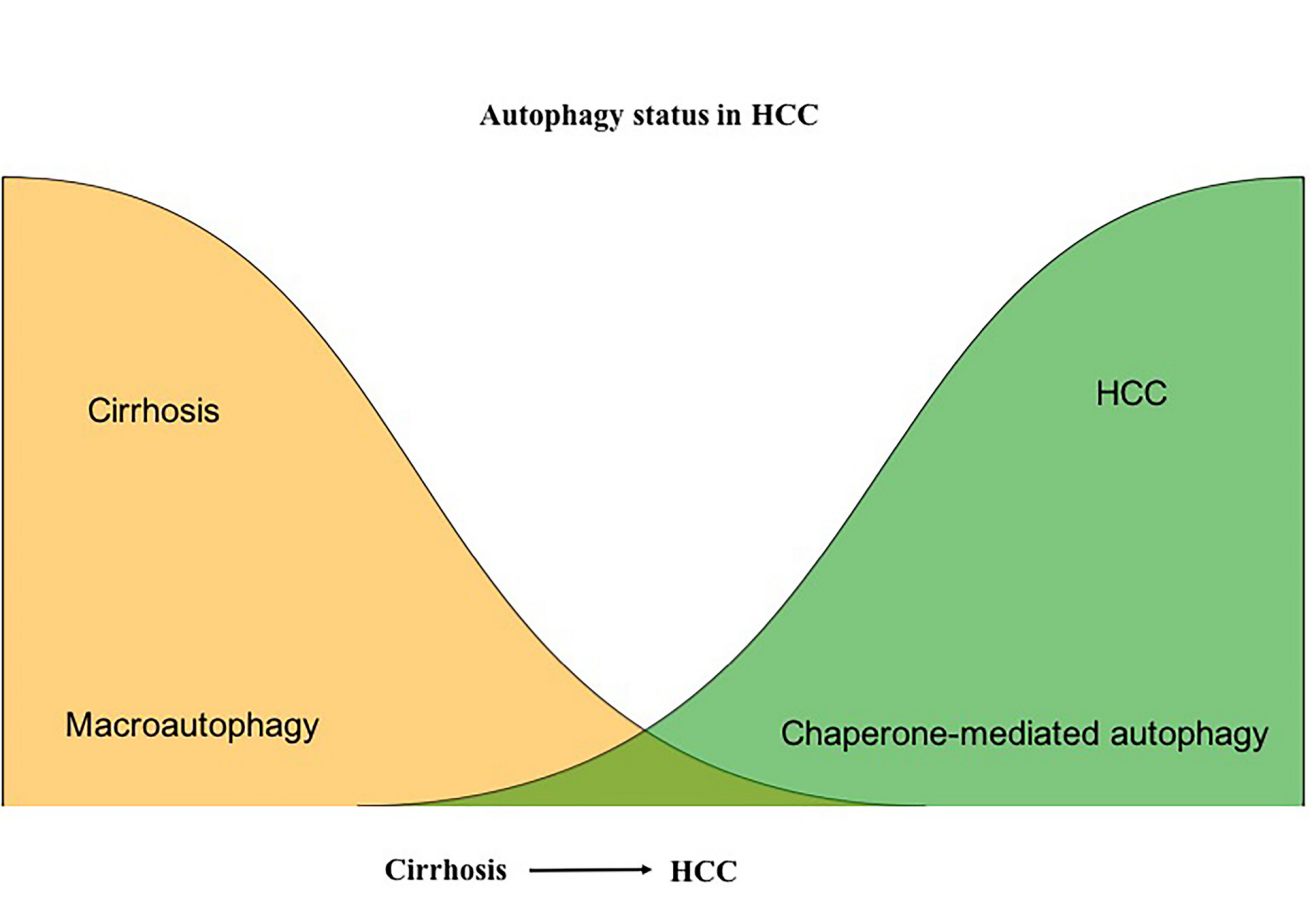
Contribution of macroautophagy and chaperone-mediated autophagy in the malignant transformation and HCC growth in cirrhotic liver Macroautophagy serves as a tumor suppressor in hepatocytes in the cirrhotic liver. Macroautophagy loss promotes HCC. CMA compensate for the impaired macroutophagy for HCC survival in the cirrhotic liver.

Our study provides evidence that the unresolved ER stress response promotes CMA as a compensatory mechanism to promote HCC survival in the context of impaired macroautophagy. Based on our results, we propose a model (Figure 14) in which impaired macroautophagy is counterbalanced by increased CMA, which may contribute to oncogenic transformation, as well as HCC growth, in the cirrhotic liver. Taken together, our studies support an important role for autophagy in malignant transformation and cancer progression. Our results showed that autophagy is needed for HCC survival since induction of autophagy promoted HCC cell growth, while autophagy inhibition by HCQ decreased proliferation and induced expression of p53 and cell cycle arrest. Importantly, we found that CMA was induced in cells with decreased macroautophagy activity, suggesting that increased CMA activity is required for HCC survival in the cirrhotic liver. Our results are also in agreement with previous reports showing that increased CMA activity is needed for tumor growth and survival in other cancers [57–59]. These findings highlight the compensatory role of CMA in promoting HCC survival in the context of liver cirrhosis when macroautophagy is impaired.

## MATERIALS AND METHODS

### Cell culture and chemicals

Huh-7.5, SK-Hep1, and HepG2 cell lines were maintained in Dulbecco's modified Eagle's medium (DMEM; Life Technologies, Carlsbad, CA), and supplemented with 2 mM L-glutamine, sodium pyruvate, nonessential amino acids, 100U/mL penicillin, 100mg/mL streptomycin and 10% fetal bovine serum (FBS). Cells were grown at 37°C in a 5% CO_2_ atmosphere within a humidified incubator with regular medium change at 3-day intervals. Hydroxychloroquine (HCQ) and Doxycycline was purchased from Sigma-Aldrich (St Louis, MO). Torin-1 was purchased from Cell Chem (Houston, TX). Antibodies specific for p62, Hsc70, BiP, p53 and Betaactin (Cell signaling, MA), an antibody to LAMP-2A was purchased from Abcam Ltd (USA); an antibody to glypican-3 was purchased from Biocare.

### Immunohistological staining

Paraffin blocks of HCCs with liver cirrhosis with viral etiologies: hepatitis C virus infection (HCV), hepatitis B virus (HBV) and non-viral etiologies: alcoholic and non-alcoholic fatty liver diseases were included in this investigation. Paraffin blocks were obtained from the Department of Pathology at the Mount Sinai Medical Center, New York and Sir Ganga Ram Hospital, Department of Pathology, New Delhi, India. Normal liver samples were obtained from NDRI. Hematoxylin and eosin (H & E)-stained sections of all specimens, including cancer and non-cancer areas of the liver tissue, were examined by two pathologists (TW and KM). Five-micron tissue sections were prepared and the slides were deparaffinized for 15 minutes at 50-60^0^ C followed by treatment with xylene twice for 5 minutes. The tissue sections were rehydrated by sequential treatment with 100%, 95% and 80% alcohol. Peroxidase quenching was carried out by incubation with 3% hydrogen peroxide and 100% methanol for 5 minutes. The slides were placed in a plastic Coplin jar with Reveal Decloaker RTU (Biocare Medical) for 25 minutes at 95°C in a steamer for heated antigen retrieval. Following this step, the slides were allowed to cool at room temperature for 20 minutes. The tissue sections were rinsed in deionized, distilled water and marked using a PAP pen. The slides were incubated with a blocking sniper (Biocare Medical) for 10 minutes and incubated with a primary antibody for 1 hour at room temperature.

Immunostaining for p62 and glypican-3 was performed using the highly sensitive micro-polymer based staining protocol (Biocare Medical). The primary antibodies used were p62 mouse monoclonal antibody (Cell signaling) (1:200 dilution) and pre-diluted antibody to glypican-3 (Biocare Medical). After the primary antibody incubation, slides were washed 3 times in Tris Buffer Saline (TBS) (pH 8.0), and incubated with a MACH 4 mouse probe (Biocare Medical, UP534) for 20 minutes and MACH 4 HRP Polymer (Biocare Medical, MRH534) for 30 minutes each, then washed 3 times using TBS. Immunostaining for Hsc70, Bip and LAMP-2A was performed using VECTASTAIN ABC kit (Vector Laboratories, Burlingame, CA) following the instructions supplied in the kit. After the staining reaction, tissue sections were treated with diaminobenzidine (DAB) chromogen (Dako Cytomation, Carpinteria, CA) for 1-5 minutes. The slides were then counterstained with hematoxylin for 30 seconds and Tacha's bluing Solution (Biocare Medical, HTBLU) for 30 seconds, dehydrated with 95% and 100% alcohol, mounted, and observed by light microscopy.

### Evaluation of immunohistochemical staining

Immunohistochemical staining of different autophagy proteins and stress markers in HCC and surrounding non-tumorous areas were examined by two pathologists (TW and KM). Scores were assigned to the intensity and percentage of positive staining in all of the slides used in this study. Scoring as follows: 0 means negative staining, 1 (weak), 2 (medium) and 3 (strong), according to a previous publication [28]. Multiplying the intensity of score and proportion of immunopositive cells (0-100%), a semiquantitative staining score, ranging from 0 to 300, was established for statistical analysis. Discrepancies were resolved by a consensus between the two pathologists using a multiheaded microscope in the Pathology Department at Tulane University Health Sciences Center. H&E-stained sections of all specimens, including cancer and non-cancer cases, were examined by the same two pathologists following the immunohistochemical evaluation.

### MTT assay

The viability of HCC cells treated with hydroxychloroquine or Torin-1 alone was measured using an MTT assay. The tetrazolium compound used in the MTT assay is reduced by the mitochondrial dehydrogenase of metabolically active cells, thus leading to the conversion of the blue tetrazolium compound into the purple precipitate formazan. This indicates the relative amount of viable cells. Quantification of formazan dye can thus be measured using a colorimetric method. HCC cells were seeded in 24-well plates at a density of 2×10^4^ cells/well in Dulbecco's Modified Eagle Medium with 10% FBS and allowed to adhere by incubation at 37°C for 24 hours. Culture medium was then replaced and cells were treated (in triplicate) with different concentrations of the drug HCQ or Torin-1. After incubating for 72 hours, cells were washed twice with PBS. Then 100 μL of MTT solution (MTT solution concentration is 5 mg/mL dissolved in PBS; thiazoyl blue tetrazolium bromide, catalog no. M5655; Sigma-Aldrich) and 900 μL of growth medium were added in each well. Cells were incubated at 37°C for 3 h. Cells were washed three times in PBS. Cells were then solubilized with 1 mL of MTT solubilization buffer (anhydrous isopropanol containing 10% Triton X-100, 0.1 N HCL) for 5 minutes. Converted dye absorbance was measured in a spectrophotometer (DU-530 UV/VIS Life Science spectrophotometer; Beckman Coulter, Brea, CA) at a wavelength of 570 nm. The percentage of cell viability was determined by comparison with untreated controls.

### Colony assay

A colony assay was performed to analyze the long-term proliferation of HCC cells in culture in the presence of macroautophagy inducer (Torin 1) and hydroxychloroquine (HCQ) (lysosome inhibitor). The HCC cells were seeded in 100 mm plates at a density of 1×10^4^ cells and allowed to attach by incubation for 24 hours at 37°C in 10 mL growth medium. After 24 hours (Day 0), media from each plate was replaced and then cells were treated with increasing concentrations of Torin 1 or HCQ. Every seven days after initial treatment (Day 7 and Day 14), plates were treated again with their corresponding drug dosages. Cells were stained with Giemsa dye when they became confluent, using a standard protocol. The media in each plate was aspirated and washed with 5 mL PBS. PBS was then aspirated and 5 mL of methanol was added to fix the cells. The methanol was then aspirated and 5 mL of a 1:5 dilution of Giemsa dye in deionized water was added to each plate. The dye was allowed to sit for 24 hours and then aspirated the next day. Plates were repeatedly washed with deionized water to get rid of background staining and then left to dry. Pictures of the plates were then taken for documentation.

### Statistical analysis

GraphPad PRISM software (San Diego, CA, USA) was used to determine the statistical significance of the differences between the experimental groups by Student's *t*-test. * *P* < 0.05, ** *P* < 0.001 and *** *P* < 0.0001.

## SUPPLEMENTARY MATERIALS FIGURES AND TABLES










