# Supplementary material for: Chaperone-mediated autophagy compensates for impaired macroautophagy in the cirrhotic liver to promote hepatocellular carcinoma
Source: Oncotarget. 2017 Mar 29;8(25):40019–36. doi: 10.18632/oncotarget.16685 (PMC5522234; doi:10.18632/oncotarget.16685)
Supplement: Supplementary file 1 [file oncotarget-08-40019-s001.pdf]

Chaperone-mediated autophagy compensates for impaired macroautophagy in the cirrhotic liver to promote hepatocellular carcinoma

Supplementary Material

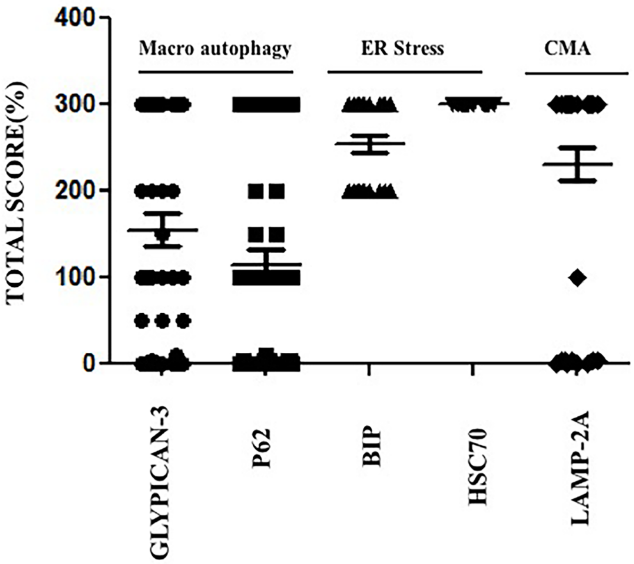

Supplementary Figure 1: Expression of Macroautophagy (p62 and glypican-3), CMA (LAMP-2A) and ER-stress (GRP78, BiP and Hsc70) in 46 cirrhotic livers with HCC. Staining intensity was semiquantified by considering the intensity of staining and the proportion of immunopositive cells. By multiplying the staining intensity score and the proportion of immunopositive cells, a staining score of 0-300 was determined.

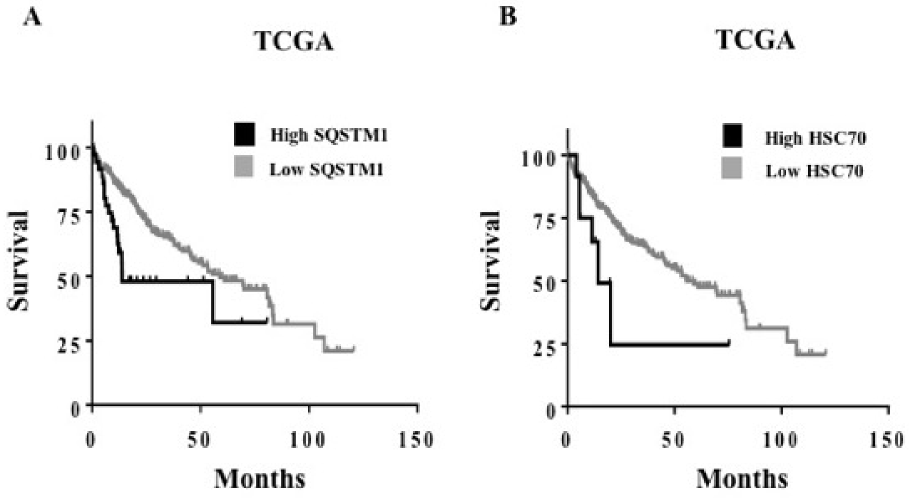

Supplementary Figure 2: The mRNA expression data of p62 and HSC70 among 442 HCC patients in TCGA online database were downloaded from cbioportal cancer genome browser. By using this data, survival curves for SQSTM1 and HSC70 were generated with help of Graphpad prism software. A. Kaplan-Meier survival curves show that increased expression of p62/SQSTM1 mRNA levels is associated with reduced survival of HCC patients. B. Kaplan-Meier survival curves show that increased expression of HSC70 mRNA levels is associated with reduced survival of HCC patients.

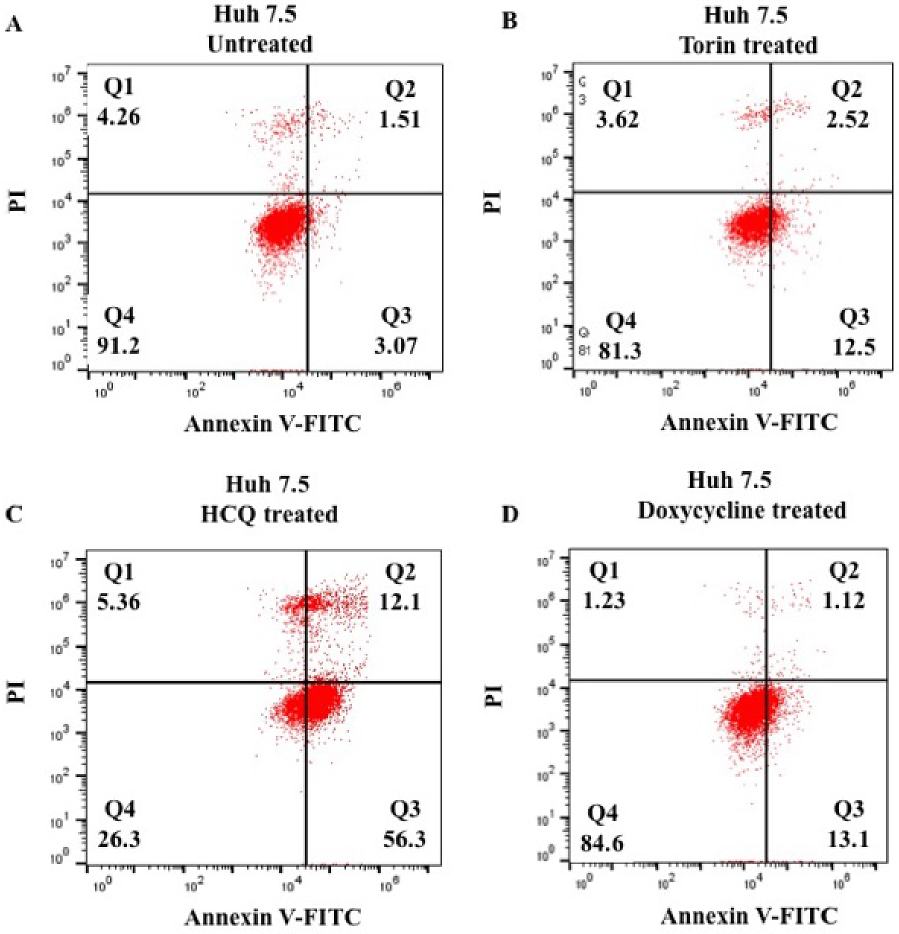

Supplementary Figure 3: Flow cytometric evaluation of apoptosis by Annexin V staining. A. Huh 7.5 cells histogram without any drug treatment. B. Huh 7.5 cells treated with Torin 1. (C). Huh 7.5 cells treated with HCQ. D. Huh 7.5 cells treated with Doxycycline.

Supplementary Table 1: Summary of p62, glypican-3, LAMP-2A, GRP78 and Hsc70 staining in HCC of viral and non-viral etiologies.
Supplementary Table 2: Summary of p62 and glypican-3 expression in 46 cirrhotic livers with HCC.
Supplementary Table 3: Summary of LAMP-2A expression in 46 cirrhotic livers with HCC.
Supplementary Table 4: Summary of Bip and HSC70 expression in 46 cirrhotic livers with HCC.

For Supplementary Tables see in Supplementary Files
