# Supplementary material for: Chaperone-mediated autophagy compensates for impaired macroautophagy in the cirrhotic liver to promote hepatocellular carcinoma
Source: Oncotarget. 2017 Mar 29;8(25):40019–36. doi: 10.18632/oncotarget.16685 (PMC5522234; doi:10.18632/oncotarget.16685)
Supplement: Supplementary file 2 [file oncotarget-08-40019-s002.docx]

| **GROUP** | **NUMBER** | **P62** | **GLYPICAN-3** | **LAMP-2A** | **HSC70** | **BIP** |
| --- | --- | --- | --- | --- | --- | --- |
| **HCV** | **16** | **15/16 (93.75%)** | **13/16 (81.25%)** | **16/16 (100%)** | **6/6 (100%)** | **6/6(100%)** |
| **HBV** | **10** | **7/10 (70%)** | **7/10 (70%)** | **9/10 (90%)** | **10/10 (100%)** | **10/10(100%)** |
| **ETOH** | **8** | **7/8 (87.5%)** | **8/8 (100%)** | **7/8 (87.5%)** | **8/8(100%)** | **8/8(100%)** |
| **NASH** | **12** | **12/12 (100%)** | **8/12 (66.6%)** | **12/12 (100%)** | **2/2 (100%)** | **2/2 (100%)** |
| **Over all** | **46** | **39/46 (84.78%)** | **36/46 (78%)** | **44/46 (95.65%)** | **26/26 (100%)** | **26/26 (100%)** |

**Supplementary Table 1 : Summary of expression of p62, Glypican-3, LAMP-2A, GRP78 and HSC70 in HCC related to viral and nonviral etiologies**
