# Supplementary material for: Chaperone-mediated autophagy compensates for impaired macroautophagy in the cirrhotic liver to promote hepatocellular carcinoma
Source: Oncotarget. 2017 Mar 29;8(25):40019–36. doi: 10.18632/oncotarget.16685 (PMC5522234; doi:10.18632/oncotarget.16685)
Supplement: Supplementary file 5 [file oncotarget-08-40019-s005.docx]

| **Supplementary Table 4: Summary of Bip and HSC70 expression in cirrhosis and HCC** | | | | | |
| --- | --- | --- | --- | --- | --- |
| **S.NO** | **Etiology** | **Bip** | | **HSC70** | |
|  |  | **HCC** | **Cirrhosis** | **HCC** | **Cirrhosis** |
|  |  | **Score** | **Score** | **Score** | **Score** |
|  |  |  |  |  |  |
| **1** | **HCV** | **+++** | **+++** | **+++** | **+++** |
| **2** | **HCV** | **+++** | **+** | **+++** | **+++** |
| **3** | **HCV** | **+++** | **+++** | **+++** | **+++** |
| **4** | **HCV** | **+++** | **+** | **+++** | **+++** |
| **5** | **HCV** | **++** | **+** | **+++** | **+++** |
| **6** | **HCV** | **++** | **+** | **+++** | **+++** |
| **7** | **HCV** | **ND** | **ND** | **ND** | **ND** |
| **8** | **HCV** | **ND** | **ND** | **ND** | **ND** |
| **9** | **HCV** | **ND** | **ND** | **ND** | **ND** |
| **10** | **HCV** | **ND** | **ND** | **ND** | **ND** |
| **11** | **HCV** | **ND** | **ND** | **ND** | **ND** |
| **12** | **HCV** | **ND** | **ND** | **ND** | **ND** |
| **13** | **HCV** | **ND** | **ND** | **ND** | **ND** |
| **14** | **HCV** | **ND** | **ND** | **ND** | **ND** |
| **15** | **HCV** | **ND** | **ND** | **ND** | **ND** |
| **16** | **HCV** | **ND** | **ND** | **ND** | **ND** |
| **17** | **HBV** | **+++** | **+** | **+++** | **+++** |
| **18** | **HBV** | **+++** | **+++** | **+++** | **+++** |
| **19** | **HBV** | **+++** | **+** | **+++** | **+++** |
| **20** | **HBV** | **++** | **+** | **+++** | **+++** |
| **21** | **HBV** | **++** | **+** | **+++** | **+++** |
| **22** | **HBV** | **+++** | **+** | **+++** | **+++** |
| **23** | **HBV** | **+++** | **++** | **+++** | **+++** |
| **24** | **HBV** | **+++** | **+** | **+++** | **+++** |
| **25** | **HBV** | **++** | **+** | **+++** | **+++** |
| **26** | **HBV** | **+++** | **+++** | **+++** | **+++** |
| **27** | **ETOH** | **+++** | **+** | **+++** | **+++** |
| **28** | **ETOH** | **++** | **+** | **+++** | **+++** |
| **29** | **ETOH** | **+++** | **+++** | **+++** | **+++** |
| **30** | **ETOH** | **++** | **+** | **+++** | **+++** |
| **31** | **ETOH** | **++** | **+** | **+++** | **+++** |
| **32** | **ETOH** | **++** | **+** | **+++** | **+++** |
| **33** | **ETOH** | **+++** | **+++** | **+++** | **+++** |
| **34** | **ETOH** | **++** | **+** | **+++** | **+++** |
| **35** | **NASH** | **ND** | **ND** | **ND** | **ND** |
| **36** | **NASH** | **ND** | **ND** | **ND** | **ND** |
| **37** | **NASH** | **ND** | **ND** | **ND** | **ND** |
| **38** | **NASH** | **ND** | **ND** | **ND** | **ND** |
| **39** | **NASH** | **ND** | **ND** | **ND** | **ND** |
| **40** | **NASH** | **ND** | **ND** | **ND** | **ND** |
| **41** | **NASH** | **ND** | **ND** | **ND** | **ND** |
| **42** | **NASH** | **ND** | **ND** | **ND** | **ND** |
| **43** | **NASH** | **ND** | **ND** | **ND** | **ND** |
| **44** | **NASH** | **ND** | **ND** | **ND** | **ND** |
| **45** | **NASH** | **+++** | **+** | **+++** | **+++** |
| **46** | **NASH** | **+++** | **+** | **+++** | **+++** |
| **(-) No staining, + Weak, ++ Medium, +++ Strong staining** | | | | | |
